# MeCP2 functions as a DNA methylation reader in immune homeostasis and cancer

**DOI:** 10.3389/freae.2026.1917394

**Published:** 2026-09-29

**Authors:** Grace C. Stroman, Clara Mellows, Kevin Pruitt

**Affiliations:** 1 Department of Pharmacology, University of North Carolina, Chapel Hill, NC, United States; 2 Lineberger Comprehensive Cancer Center, University of North Carolina, Chapel Hill, NC, United States

**Keywords:** cancer, DNA methylation, epigenetics, immune system, MeCP2, neurodevelopment, rett syndrome

## Abstract

DNA methylation is a key epigenetic mechanism that mediates gene expression and informs cellular identity. Methyl-CpG binding protein 2 (MeCP2) is a protein capable of recognizing and interpreting the methylated DNA code to bring about phenotypic outcomes through co-factor interactions. Altered DNA methylation patterns and MeCP2 function, therefore, disrupt cellular epigenetic homeostasis and contribute to human diseases. MeCP2 loss-of-function or amplification events are responsible for Rett syndrome and *MECP2* duplication syndrome, respectively, where patients present with multisystem pathophysiology. Growing evidence suggests that MeCP2 contributes to immune dysregulation in these syndromes and is a key epigenetic regulator in additional inflammatory conditions. Research in past decades has functionally linked MeCP2 to tumorigenesis across cancer types, where it can support and inhibit oncogenic properties in a contextual manner. The precise mechanistic role of MeCP2 in non-neuronal and cancer models is underexplored. Here, we summarize the diverse epigenetic functionalities of MeCP2 influenced by its molecular structure, regulation, and co-factor interactions and discuss how MeCP2 regulates immune homeostasis and tumorigenesis.

## 1 Introduction

Patterns of gene expression give rise to cellular identity and behavior in response to external cues in an ever-changing cellular milieu. Hence, the precise, tunable, and reversible regulation of gene expression is critical for cellular adaptation and survival. Epigenetic regulation allows for somatically heritable gene expression independent of the DNA sequence. Epigenetic regulation is “encoded” by chemical DNA and histone modifications, nucleosome remodeling, and non-coding RNAs. Crosstalk between these regulatory layers provides highly context-dependent, precise, and heritable chromatin organization and transcription profiles. Remarkably, the orchestration of multiple layers of epigenetic regulation enables unique cell types to arise from cell-type and tissue-dependent expression of identical genetic material throughout development (Pruitt, 2016). Epigenetic dysregulation can result in metabolic and autoimmune diseases, neurological disorders, and cancer (Farsetti et al., 2023; Yu et al., 2024). Recently, the role of epigenetics in cancer has been refined through the proposal of six cancer hallmarks (Esteller et al., 2024).

Epigenetic states are in constant flux due to the dynamic and reversible covalent modifications to DNA and histones. DNA methylation occurs at the fifth carbon of cytosine (5 mC) residues in prokaryote and eukaryote systems (Mattei et al., 2022). Although cytosine methylation predominately occurs at CpG dinucleotides, non-CpG methylation is observed in unique cell types such as stem cells, neurons, and glial cells (Jang et al., 2017). DNA methylation is a dynamic process whereby “writer,” “reader,” and “eraser” protein machinery shape the epigenome. The “writers” are DNA methyltransferases which deposit methyl groups from S-adenyl methionine (SAM) to cytosine during DNA replication (i.e., DNMT1) or unmodified DNA *de novo* (i.e., DNMT3a and DNMT3b). In turn, “eraser” proteins can demethylate DNA through a series of hydroxymethylation (TET1, TET2, TET3), deamination (AID, APOBEC), and base excision repair (TDG, SMUG1) biochemical steps, as seen in global demethylation events during development (Moore et al., 2013).

DNA methylation “readers” are proteins that bind to methylated DNA and interact with transcription, splicing, and chromatin factors to translate the DNA methylation code into epigenetic instructions (Moore et al., 2013). These readers are broadly classified into the methyl CpG binding domain (MBD), Set and RING-associated domain, and methyl-CpG binding zinc finger protein families (Mahmood and Rabbani, 2019). Within the MBD family, there are seven proteins: MeCP2, MBD1, MBD2, MBD3, MBD4, MBD5, and MBD6. Although this protein family contributes to various human diseases and disorders, research efforts have historically focused on DNMTs (Ginder and Williams, 2018). This led to the development of hypomethylating agents and Food and Drug Administration approval of 5-Azacytidine as a DNMT inhibitor for oncology indications (Itzykson et al., 2015; Scott, 2016). This review will focus on the clinical relevance of MeCP2, which has a well-established role in neuropsychiatric and neurodevelopmental disorders with multisystem pathophysiology and an emerging role in cancer (Nejati-Koshki et al., 2023; Akaba and Takahashi, 2025; Liu et al., 2025a; Liu et al., 2025b). MeCP2 is a versatile and dosage-sensitive X-linked gene that confers diverse epigenome regulation through finely nuanced co-factor dynamics (Liu et al., 2025b). Here, we summarize the current knowledge of MeCP2 structure, regulation, and function in human diseases and disorders beyond the neurological setting with a special focus on cancer.

## 2 MeCP2 structure, regulation, and function

### 2.1 Structure

MeCP2 was the first DNA methylation reader identified, and its discovery established a new protein family defined by the presence of a MBD (NCBI Conserved Domain Database ID: cl00110) (Lewis et al., 1992). This MBD sequence later identified an additional ten proteins classified into the MeCP2-MBD, histone methyltransferase-MBD, and histone acetyltransferase-MBD families (Hendrich and Bird, 1998). MeCP2 is an intrinsically disordered protein with six functional domains shared across the MeCP2-MBD family. These include the N-terminal domain (NTD), methyl binding domain (MBD), intervening domain (ID), transcription repression domain (TRD) (also known as the Nuclear Receptor Corepressor (NCoR) interaction domain (NID)), nuclear localization sequence (NLS), RNA-binding domain (RBD), AT-hook motifs, and C-terminal domain (CTD) (Ghosh et al., 2010). The MBD is required for interacting with methylated DNA and is the most structurally defined region (Nan et al., 1993; Ghosh et al., 2010). The NTD, ID, and TRD regulate the MBD’s affinity for methylated DNA. MeCP2 can bind to the minor groove of AT-rich DNA via its AT-hooks. The TRD is essential for nucleosome binding, contains the NLS for nuclear import, and a newly recognized non-canonical RBD sufficient for mediating RNA interactions (Chua et al., 2024; Good et al., 2026). The TRD and MBD also recruit the NCoR1/SMRT co-repressor complex, one of the best characterized MeCP2 protein interactors (Lyst et al., 2013). Finally, the CTD contributes to DNA and chromatin binding abilities and can mediate nucleosome compaction. Altogether, these domains serve to mediate MeCP2 binding to DNA, protein, and RNA co-factors and modulate local and global chromatin states that will be described in the next section.

### 2.2 MeCP2 mRNA and protein regulation

*MECP2* is an X-linked gene essential for postnatal brain development. Although *MECP2* is expressed in all tissues, it is especially prevalent in neurons (Shahbazian et al., 2002). It plays an essential and unique role amongst the MBD family in maintaining neuron health in the central nervous system. Depletion of *Mecp2* in the mouse brain results in severe neurological symptoms and death between 6 to 12 weeks, a phenotype not observed with the disruption of other MBD proteins (Tillotson and Bird, 2020). Located on the X chromosome, MeCP2 is subject to random X chromosome inactivation established in the early embryo (Adler et al., 1995). Proper expression and function of MeCP2 are key for brain health and disruptions contribute to X-linked genetic disorders, such as Rett Syndrome, that will be elaborated on in a later section.

Although our understanding of *MECP2* transcriptional regulation is limited, *MECP2* undergoes post-transcriptional regulation via various processes. At the transcriptional level, Monocyte enhancer factor 2C (MEF2C) positively regulates MeCP2 E-box promoter activity while HMGN1 perturbs transcriptional initiation (Zweier et al., 2010; Abuhatzira et al., 2011). Overexpression of *MECP2* engages in an autoinhibitory feedback mechanism to maintain expression equilibrium (Lockman et al., 2024). Interestingly, MEF2C is a key regulator of immune cell development, inflammatory responses, and metabolic control within the immune system (Zhao X. et al., 2022; Di Giorgio et al., 2021; Li J. et al., 2024). In addition, pathogenic variants in MEF2C phenocopies hallmarks of Rett Syndrome (Lambert et al., 2012). Despite these connections, it is unclear if disruptions in the MEF2C-MeCP2 signaling axis contribute to MeCP2-mediated immune system dysregulation that will be addressed in a later section. At the post-transcriptional level, *MECP2* undergoes alternative splicing to generate two isoforms with unique functionalities. The MeCP2-E1 (e1) and MeCP2-E2 (e2) isoforms share the same functional domains but differ in their N terminus due to alternative exon 2 splicing (Kriaucionis and Bird, 2004; Mnatzakanian et al., 2004). Additionally, the upstream e1 open reading frame increases e1 and reduces e2 translational abundance (Kriaucionis and Bird, 2004). Despite this, the isoforms vary in their tissue expression where e1 is the major isoform in the brain and e2 is the predominate isoform in the placenta, liver, and skeletal muscle (Mnatzakanian et al., 2004). Recently, researchers identified an alternative splicing mechanism governing e2 abundance in mouse cell lines. In their model, exon 2 retention is driven by an SRSF9-bound exonic splicing enhancer within exon 2, along with strong splice sites adjacent to exon 2 (Oshizuki et al., 2025). Differential isoform expression dictates the severity of a MeCP2-driven neurodevelopmental disorder that will be discussed in a later section (Itoh et al., 2012; Yasui et al., 2014). As a result, modulating *MECP2* splicing events is considered a therapeutic strategy (Tirumala et al., 2026). Another avenue of post-transcriptional regulation is controlled by four alternative polyadenylation sites which determine protein and microRNA binding sites for *MECP2* mRNA regulation (Rodrigues et al., 2016). Extensive work in the field has identified numerous microRNAs (miRNAs) that regulate mRNA availability. These include miR-7b, miR-132/212, miR-181, miR-194-3p, miR-373, miR-422a, and miR-483-5p. These miRNAs explain broad and tissue-specific modes of *MECP2* regulation and link *MECP2* to Alzheimer’s disease, ischemic injury, human immunodeficiency virus (HIV), osteoarthritis, adipogenesis, and breast cancer (Klein et al., 2007; Hutchison et al., 2013; Chen et al., 2014; Xie et al., 2019; Zhou et al., 2020; Giuliani et al., 2023; Wang et al., 2026; Du et al., 2026). Finally, YTHDF1 enhances *MECP2* mRNA stability by binding to N^6^-methyladenosine modified *MECP2* mRNA (Tu et al., 2025). These findings demonstrate the breadth of *MECP2* mRNA regulation, which has implications for MeCP2-driven neuropathologies and cancer but remain underdeveloped.

Post-translational modifications (PTMs) further regulate MeCP2 protein function and stability although our understanding is primarily limited to neuron models given the importance of MeCP2 in postnatal brain development. While significantly more work is needed to clearly resolve how MeCP2 switches between transcription, splicing, and chromatin regulation, emerging evidence suggests that MeCP2 PTMs could serve as this important regulatory switch to dynamically contextualize its molecular role. Phosphorylation is the best characterized MeCP2 PTM and is reviewed elsewhere (Bellini et al., 2014; Tillotson and Bird, 2020). In summary, MeCP2 phosphorylation alters its interactions with DNA, the NCoR1 complex, and heterochromatin in a manner dependent or independent of neuronal activity (Ebert et al., 2013; Stefanelli et al., 2016; D’Annessa et al., 2018; Schmidt et al., 2022). In the brain, phosphorylation of MeCP2 mediates downstream microglial proinflammatory responses, synapse and striatum maturation, and stress resilience (Wittrahm et al., 2021; Huang et al., 2022; Tzeng et al., 2023; Varela-Andrés et al., 2026). Outside of the brain, phosphorylation of MeCP2 influences liver cell proliferation and fibrosis (Moran-Salvador et al., 2019). Phosphorylation provides the most comprehensive view of how PTMs fine-tune MeCP2 interactions and downstream phenotypes, highlighting the importance of PTM characterization.

MeCP2 acetylation provides an additional layer of regulation. Choudhary and colleagues initially discovered MeCP2 acetylation at K461 and K499 using tandem mass spectrometry and deacetylase inhibitors in mixed linage cell models, which were later validated (Choudhary et al., 2009; Hornbeck et al., 2012; Zocchi and Sassone-Corsi, 2012). Neuronal cells are uniquely linked with K305, K307, and K321 acetylation, and additional residues were identified in cancer cells (Gonzales et al., 2012; Pandey et al., 2015; Castro-Piedras et al., 2020). The only biological validation was carried out by Pandey and colleagues who found that K171 acetylation mediates MeCP2 interactions with ATRX, HP1α, and HDAC1 using SIRT1 inhibitors (Pandey et al., 2015). Since K135 is mutated in Rett Syndrome and acetylation neutralizes the residue’s positive charge, it is of great interest how its post-translational regulation influences MeCP2 function (Pandey et al., 2015). While the substitution of K>Q at K135 was shown to not alter MeCP2 nuclear localization, it remains unknown whether acetylation of this residue, or any of the other 21 acetylated lysines mapped, serve as a molecular switch specifying transcription repression vs activation or other aspects of MeCP2 function (Castro-Piedras et al., 2020). The role of MeCP2 acetylation is in its infancy, and further investigation is anticipated to deepen our understanding of its contextual functions.

Similar to acetylation, MeCP2 ubiquitination and SUMOylation residues have been identified, but functional biological validation remains underexplored (Kalani et al., 2023). Gonzales identified multiple ubiquitination sites by tandem mass spectrometry in neuronal cells which were corroborated in HEK-293T cells (Wagner et al., 2011; Gonzales et al., 2012). These ubiquitination moieties are deposited by E3 ubiquitin ligases including RNF4, DCAF13, NEDD4, and HERC2 (Wang, 2014; Ren et al., 2024; Yang et al., 2024; Zhou et al., 2025). Besides the known HERC2-mediated K48 polyubiquitination site, the MeCP2 target residues of these E3 ligases remain unexplored (Zhou et al., 2025). On the other hand, USP15 is the only reported deubiquitinase and promotes MeCP2 accumulation under hypoxic conditions (Zhang Z. et al., 2025). Ubiquitination occurs on both K130 and K135 residues within the MBD suggesting that this PTM could potentially regulate MeCP2 selectivity in binding to methylated vs. unmethylated DNA (Gonzales et al., 2012). As the K135 residues can undergo acetylation or ubiquitination, this residue may be subject to competing activating and inhibitory PTM regulation that remains elusive. Regarding SUMOlyation, the K223 residue of MeCP2 is critical for HDAC1/2 recruitment and transcriptional repression in neurons (Cheng J. et al., 2014). When SUMOylated at K412 (human K426) by the E3 ubiquitin ligase Pias1, Mecp2 no longer interacts with the transcription factor Creb, resulting in altered transcription (Tai et al., 2016). A comprehensive map of putative SUMOlyation sites remains a gap in the field, and the biological significance of these PTMs remains an ongoing effort. As ubiquitination adds complex electrostatic properties via a globular ubiquitin moiety Several of these residues undergo missense mutations in MeCP2 pathologies and disrupted PTMs may have clinical implications.

Several understudied MeCP2 PTMs include methylation, lactylation, and glycosylation. As of now, several methylation sites have been reported (Jung et al., 2008; Guo A. et al., 2014). Recent work by Schmidt and colleagues identified MeCP2 undergoes R91, R106, R167, and R162 methylation in the brain. Methylation of R91 has been shown to modulate heterochromatin organization while R106 methylation is critical for regulating DNA binding and heterochromatin clustering (Schmidt et al., 2022). Future studies could focus on the R106 and R162 sites as disruptions may contribute to MeCP2 pathology or RBD function, respectively. MeCP2 is also subject to lactylation modification. Researchers identified that exercise-induced K271 lactylation mediates MeCP2 DNA binding, gene expression, chromatin accessibility, and macrophage polarization in an atherosclerosis model underscoring how PTMs contextualize MeCP2 molecular functions to direct immune cell biology (Wang et al., 2023; Chen et al., 2024). Additionally, K210 and K249 lactylation results in suppression of pro-apoptosis genes and may improve ischemic stroke recovery (Sun et al., 2025). Finally, only a handful of O-GlcNAcylation MeCP2 moieties have been identified in the mouse brain (Trinidad et al., 2012; Wang et al., 2010; Cheng et al., 2021). Altering the T203 residue suppresses *Bdnf* transcription during neuronal activation and impairs neural development (Cheng et al., 2021). Altogether, these findings demonstrate how PTMs to contextualize MeCP2 function and hold potential clinical significance.

### 2.3 MeCP2-DNA interactions

MeCP2 was first described as a transcriptional repressor through its interactions with 5-methylcytosine (5 mC) CpG (Lewis et al., 1992). Recent binding studies reveal how MeCP2 binds to DNA in a manner sensitive to local nucleic acid topology in addition to cytosine methylation affinity (Melikishvili et al., 2026). Later studies reveal that MeCP2 recognizes 5-hydroxymethylcytosine (5hmC), non-CpG methylation, and non-methylated DNA. Interestingly, MeCP2 binds to 5mC and 5hmC with similar affinities but shows a preference for 5-hydroxymethyl CpA *in vitro* and *in vivo* (Mellén et al., 2012; Khrapunov et al., 2014; Gabel et al., 2015; Kinde et al., 2016). Additionally, the A/T bases adjacent to methylated DNA dictate MeCP2 affinity for select sites likely due to its AT-hook motif (Klose et al., 2005), buthe biological significance of this binding preference remains unexplored. Non-CpG methylation is unique to stem cells, neurons, and glial cells, and is relevant when studying MeCP2-driven neurological pathologies (Varley et al., 2013; Guo J. et al., 2014; Clemens et al., 2020). Lastly, the field has documented MeCP2 promotes transcriptional activation when localized to non-methylated DNA through single-molecule and *in vitro* studies(Yasui et al., 2007; Chahrour et al., 2008; Liu et al., 2024). Since its discovery, the field continues to develop a deeper understanding of the complex interactions between MeCP2 and nucleic acids and cytosine modifications influence this dynamic process. While its importance for MeCP2-driven neurological pathologies has been investigated more extensively, the implications of this context-dependent regulation and binding remains poorly understood in cancer.

### 2.4 MeCP2-protein interactions

MeCP2 interacts with protein co-factors to execute its broad epigenetic reader functions. Initially thought to act as a transcriptional repressor through co-repressor complex recruitment, the protein interactome of MeCP2 now encompasses over 40 diverse partners which further expands our appreciation for its involvement in transcription, RNA processing, DNA stability, and chromatin organization (Figure 1; Table 1) (Jones et al., 1998; Tillotson and Bird, 2020). Originally hypothesized to be a global repressor, subsequent microarray and RNA-Sequencing (RNA-Seq) experiments indicate that MeCP2 is a subtle brain transcriptional regulator dependent on cell type and species (Tudor et al., 2002; Trostle et al., 2023; Gioiosa et al., 2025). By partnering with NCoR1/SMRT, c-Ski, SIN3A, CoREST, and CBF1 co-repressor complexes, MeCP2 encourages transcriptional repression through regional histone PTM modifications (Nan et al., 1998; Kokura et al., 2001; Lunyak et al., 2002; Mann et al., 2007). Gene silencing also arises through MeCP2’s recruitment of TFIIB, which interferes with transcription preinitiation complex assembly, and inhibition of TOP2β activity at methylated long genes in the brain (Kaludov and Wolffe, 2000; Nettles et al., 2023). On the other hand, MeCP2 facilitates transcription via interactions with RNA pol II, the super elongation complex, and transcriptional activators (i.e., CREB1, TCF20, LEDGF) (Chahrour et al., 2008; Leoh et al., 2012; Zhou et al., 2022; Liu et al., 2024; Sonn et al., 2025). How DNA methylation influences MeCP2 localization with these transcriptional regulators is not understood. These additional protein interactions reveal the contextual role of MeCP2 during gene activation and repression.

**FIGURE 1:**
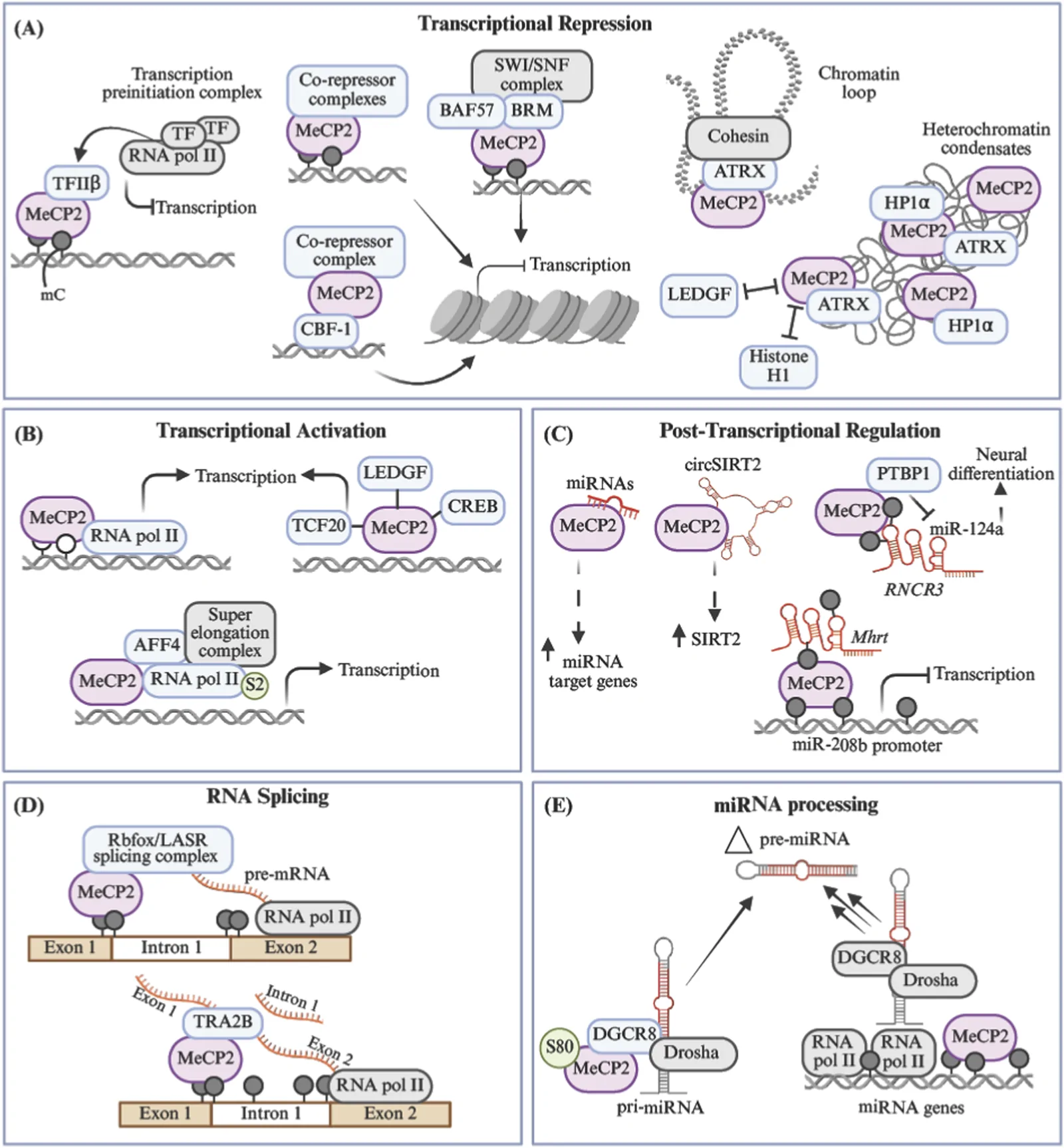
The molecular functions of MeCP2-co-factor interactions. **(A)** MeCP2 imparts transcriptional silencing by inhibiting the formation of the transcription preinitiation complex through the sequestration of TFIIβ, one of its core components. MeCP2 also recruits co-repressor complexes at methylated cytosines (mC) or select CBF-1 sites and components of the SWI/SNF nucleosome remodeling complex. These multimeric protein complexes enforce a transcriptionally inaccessible regional state through selective histone deacetylation and demethylation (co-repressor) or nucleosome sliding (SWI/SNF). Higher level chromatin compaction occurs through MeCP2’s recruitment of ARTX to suppress imprinted genes through chromatin loops or selective recruitment of heterochromatin factors (ATRX, HP1α) and antagonism with LEDGF and histone H1 to bring about heterochromatin condensate formation. **(B)** MeCP2 facilitates transcriptional activation by localizing RNA pol II to hypomethylated regions and partnering with transcription factors such as TCF20, LEDGF, and CREB. MeCP2 also partners with AFF4 as part of the super elongation complex. In these ways, MeCP2 contributes to RNA pol II initiation and elongation. **(C)** MeCP2 indirectly contributes to post-translational regulation via its RNA co-factors. The interaction between MeCP2 and miRNAs or circRNAs may interfere with the co-factor’s target gene inhibition resulting in elevated target gene or protein levels. Additionally, when bound to the methylated lncRNA *RNCR3*, MeCP2 recruits PTBP1 to prevent pri-miRNA processing and expression of the internally encoded miR-124a to inform neural differentiation. MeCP2 also indirectly informs sex-specific heart development through the suppression of miR-208b when engaged with its methylated promoter and the methylated lncRNA *Mhrt*. **(D)** The presence of DNA methylation and MeCP2 influences RNA slicing events through several approaches. It is postulated that RNA pol II pausing within gene bodies enriched in DNA methylation and MeCP2 allows more time for spliceosome inclusion of alternatively spliced regions. MeCP2-mediates pre-mRNA splicing as a component of the Rbfox/LASR splicing complex and interacts with TRA2B which promotes intron exclusion. **(E)** MeCP2 contributes to miRNA processing to indirectly regulate the post-translational landscape. When phosphorylated at S80, MeCP2 binds to DGCR8 to influence primary-miRNA (pri-miRNA) processing by Drosha. When occupying methylated miRNA genes, MeCP2 hinders RNA pol II efficiency and allows for additional DGCR8-Drosha processing. Acronyms: transcription factor (TF). Created in https://BioRender.com.

**TABLE 1:** MeCP2 protein interactors.

Function	Interactor	Method	Model	Citation
DNA helicase	TOP2β	Co-immunoprecipitation	Mouse cortical neurons	(Nettles et al., 2023)
		Co-immunoprecipitation	Mouse brain	(Long et al., 2011)
Chromatin regulators	HP1	Co-immunoprecipitation, pull-down	HEK293-EBNA cells	(Agarwal et al., 2007)
	DNMT1	Co-immunoprecipitation, pull-down	HEK293T cells	(Kimura and Shiota, 2003)
	SIRT1	Co-immunoprecipitation	HEK293 cells	(Pandey et al., 2015)
	PRMT6	Co-immunoprecipitation	Min6 cells	(Dhawan et al., 2011)
	G9a	Co-immunoprecipitation	Neonatal mouse brain	(Subbanna et al., 2014)
	HLCS	Co-immunoprecipitation, limited proteolysis assay	HEK293 cells	(Xue et al., 2013)
	LANA	Co-immunoprecipitation, colocalization	HeLa, NIH 3T3 cells	(Krithivas et al., 2002)
		Co-immunoprecipitation, co-localization, pull-down	HEK293T cells	(Matsumura et al., 2010)
		Co-immunoprecipitation, yeast two-hybrid screen	HEK293T cells	(Verma et al., 2013)
	SUV39H1	Co-immunoprecipitation	Rat-1 cells	(Lunyak et al., 2002)
	ATRX	Yeast two-hybrid screen, pull-down, co-immunoprecipitation	Mouse L cells	(Nan et al., 2007)
	SMARCB1	Glycerol-gradient sedimentation, pull-down	NIH3T3 cells	(Harikrishnan et al., 2005)
		Co-immunoprecipitation	HEK293 cells	(Luoni et al., 2026)
	BRG-1	Co-immunoprecipitation	Mouse brain	(Maxwell et al., 2013)
	BAF57	Glycerol-gradient sedimentation, pull-down	NIH3T3 cells	(Harikrishnan et al., 2005)
	Brm	Glycerol-gradient sedimentation, co-immunoprecipitation, pull-down, colocalization	NIH3T3 cells	(Harikrishnan et al., 2005; Hu et al., 2006)
		Co-immunoprecipitation	Mouse brain	(Maxwell et al., 2013)
	PHF14	BioID-mass spectrometry, co-immunoprecipitation, co-localization	Rat primary neurons, mouse cortex, mouse 3T3 fibroblasts, HEK293T cells	(Zhou et al., 2022)
RNA processing factors	YB1	Co-immunoprecipitation, affinity purification-mass spectrometry, pull-down	HeLa, Neuro2A, CEM-CCRF cells, mouse brain	(Young et al., 2005)
	FBP11	Co-immunoprecipitation, pull-down	HEK293 cells	(Buschdorf and Strätling, 2004)
	HYPC	Co-immunoprecipitation, pull-down	HEK293 cells	(Buschdorf and Strätling, 2004)
	Rbfox/LASR complex	Co-immunoprecipitation, affinity purification-mass spectrometry	HEK293T cells and mouse brain	(Jiang et al., 2021)
	DHX9	Co-immunoprecipitation	Mouse brain	(Maxwell et al., 2013)
		Co-immunoprecipitation	Mouse brain	(Li et al., 2016)
	PRP8	Co-immunoprecipitation	Mouse brain	(Maxwell et al., 2013)
	PRPF3	Co-fractionation, co-immunoprecipitation, pull-down	Rat brain, HT22 cells	(Long et al., 2011)
	DGCR8	Pull-down, co-immunoprecipitation	HEK293	(Cheng T. et al., 2014)
	TRA2B	Affinity purification-mass spectrometry	Mouse promyelocyte and granulocyte cells	(Wong et al., 2017)
Transcription regulators	RNA pol II	Co-immunoprecipitation, affinity purification-mass spectrometry, pull-down	Human and mouse neurons, HEK293T cells	(Liu et al., 2024)
		Co-immunoprecipitation	HEK293T cells, mouse cortex	(Sonn et al., 2025)
	AFF4	Bimolecular fluorescence complementation, co-immunoprecipitation	HEK293T cell, mouse cortex	(Sonn et al., 2025)
	YY1	Pull-down, co-immunoprecipitation	HEK293 cells	(Forlani et al., 2010)
	CREB1	Affinity purification-mass spectrometry, co-immunoprecipitation	Mouse brain	(Chahrour et al., 2008)
	SP3	Co-immunoprecipitation	P19 cells	(Hwang et al., 2010)
	TFIIB	Pull-down	*in vitro*	(Kaludov and Wolffe, 2000)
	SPI1/PU.1	Pull-down, co-immunoprecipitation	MEL cells	(Suzuki et al., 2003)
	TCF20	BioID-mass spectrometry, co-immunoprecipitation, co-localization	Rat primary neurons, mouse cortex, mouse 3T3 fibroblasts, HEK293T cells	(Zhou et al., 2022)
	LEDGF	Transcription factor protein arrays, pull-down, AlphaScreen, co-immunoprecipitation, colocalization	HEK293T, U2OS, PC3 cells	(Leoh et al., 2012)
		Co-immunoprecipitation	Mouse brain	(Li et al., 2016)
		Co-immunoprecipitation, pull-down, AlphaScreen	HEK293T cells	(Lesire et al., 2025)
	CXXC5	BioID-mass spectrometry, co-immunoprecipitation, proximity ligation assay	HEK293 cells	(Ayaz et al., 2021)
	CBF1	Co-immunoprecipitation	Myofibroblasts cells	(Mann et al., 2007)
Co-repressor complexes	c-Ski	Pull-down, co-immunoprecipitation	HEK293T cells	(Kokura et al., 2001)
	NCoR1 via TBLR1	Affinity purification-mass spectrometry, co-immunoprecipitation	Mouse brain, HeLa cells	(Lyst et al., 2013)
		Pull-down	*in vitro*	(Kokura et al., 2001)
	SMRT and HDAC3	Affinity purification-mass spectrometry, co-immunoprecipitation	Mouse brain, HeLa cells	(Lyst et al., 2013)
	SIN3A	Gel-filtration chromatography, ion-exchange chromatography, co-immunoprecipitation	*Xenopus* oocyte	(Jones et al., 1998)
		Pull-down	HeLa cells, rat brain	(Nan et al., 1998)
	CoREST	Co-immunoprecipitation	Rat-1 cells	(Lunyak et al., 2002)

Subsequent sequencing analysis and protein interactome mapping confirmed that MeCP2 mediates RNA processing (Figure 1). Ablating MeCP2 expression in mouse and human models identified aberrant alternative splicing events including skipped exons, mutually exclusive exons, alternative 3′ or 5′ splice sites, and retained introns which impact mouse memory and learning. More specifically, the presence of DNA methylation and MeCP2 within alternatively spliced exons or near intron splice junctions can mediate exon or intron retention, respectively (Maunakea et al., 2013; Cheng et al., 2017; Osenberg et al., 2018; Brito et al., 2020). The majority of alternatively spliced genes were found to have similar gene expression levels between wild type and knockout indicating independent transcript and splicing MeCP2 regulatory pathways (Li et al., 2016; Brito et al., 2020). Additionally, It was suggested that MeCP2 occupancy and/or histone hypoacetylation at specific methylated exons and introns contribute to RNA pol II pausing and allows time for spliceosome inclusion of alternatively spliced regions (Maunakea et al., 2013; Wong et al., 2017; Cholewa-Waclaw et al., 2019). MeCP2 may regulate alternative splicing through its interactions with pre-mRNA splicing factors such as YB-1, FBP11, HYPC, LEDGF, DHX9, PRPF3, and TRA2B (Buschdorf and Strätling, 2004; Young et al., 2005; Long et al., 2011; Li et al., 2016; Cheng et al., 2017; Wong et al., 2017). Although it is not clear if and how all of these interactions directly modulate splicing. In the case of TRA2B, it interacts with MeCP2 at select transcripts to prevent intron retention (Wong et al., 2017). Furthermore, as a member of the multi-subunit Rbfox/LASR splicing regulator, MeCP2 depletion perturbs complex formation and pre-mRNA target binding. This leads to aberrant splicing for a significant percent of target genes, including those critical for synapse dynamics (Jiang et al., 2021). In contrast to the present evidence, a reanalysis of published transcriptomic datasets found minimal alternative splicing events with differential MeCP2 expression, thus questioning the functional significance of MeCP2 in splicing regulation (Chhatbar et al., 2020). However, a more recent integrated alternative splicing analysis using 100 human and 130 mouse *MECP2* mutant datasets continued to find significant splicing dysregulation (Gioiosa et al., 2025). This analysis identified 20 genes that were differentially spliced in at least three datasets, suggesting that MeCP2 splicing regulation may be species and cell type dependent and requires careful interpretation. Beyond splicing, MeCP2 influences primary miRNA (pri-miRNA) processing through interactions with DGCR8 as part of the Drosha complex. This interaction is dependent on S80 phosphorylation status and alters mTOR signaling and neural development (Cheng T. et al., 2014; Tsujimura et al., 2015). Building upon these findings, MeCP2 occupancy at methylated miRNA loci augments RNA pol II elongation and enhances miRNA processing by Drosha while unmethylated miRNAs remain unaltered (Glaich et al., 2019). These studies demonstrate a clear role for MeCP2 in RNA metabolism which future studies can continue to probe mechanistically.

MeCP2 has also been implicated in maintaining DNA stability through its protein co-factors, although much remains mechanistically scarce. Ablation of MeCP2 can trigger senescence and lead to the accumulation of DNA damage foci, R-loops, double-strand DNA breaks, and reactivation of repetitive and transposable elements (Squillaro et al., 2010; Skene et al., 2010; Muotri et al., 2010; Marchena-Cruz et al., 2023). These observations were further validated when a *Mecp2* null mouse screen identified that DNA damage response gene mutations could reverse *Mecp2* neuropathological symptoms (Enikanolaiye et al., 2020). MeCP2 can inhibit TOP2β, a neuron-specific type II topoisomerase important for releasing torsional stress, to influence the expression of long genes but it remains unknown if this interaction influences DNA stability (Enikanolaiye et al., 2020). Alternatively, MeCP2 may influence genome stability through unknown RNA co-factors or guide repair through its interaction with ATRX, an RNA-binding protein known to promote DNA repair and inhibit R-loop formation and transposable element expression (Aguilera and López-Contreras, 2023). Understanding how protein and RNA co-factors execute the emerging genome-protective function of MeCP2 in neuronal and non-neuronal contexts remains to be explored at the molecular level.

MeCP2 orchestrates higher-order chromatin remodeling by bridging epigenetic layers from DNA methylation to nucleosomes and chromatin organization (Figure 1). At the level of DNA, MeCP2 interacts with DNMT1 to maintain DNA methylation patterns during DNA synthesis (Kimura and Shiota, 2003). MeCP2 can also bind directly to histones (i.e., H2A, H2B, H3, H4, H2A.X) and histone PTMs (i.e., H3K4me3, H3K9me2/3, H3K27me3, H3K36me3) as shown through biochemical and *in vitro* binding studies (Lunyak et al., 2002; Thambirajah et al., 2012; Lee et al., 2020; Ortega-Alarcon et al., 2024). These studies indicate how MeCP2 may facilitate transcriptional repression (H3K9me3 and H3K27me3) or activation (H3K4me3 and H3K36me3) through contextual interactions. MeCP2 can influence histone PTMs, transcription, and nucleosome remodeling indirectly by partnering with co-activator/repressor complexes in brain models as previously alluded to (Nan et al., 1998; Jones et al., 1998; Lunyak et al., 2002; Mann et al., 2007; Lyst et al., 2013). More recent studies confirm that MeCP2 is essential for recruiting co-repressor complexes to suppress gene expression beyond the brain in hepatocyte, lung, and bone models (Kyle et al., 2016; Vashi et al., 2021; Zhang Z. et al., 2024). MeCP2 also influences higher level nucleosome remodeling, chromatin looping, and potentially condensate formation. When associated with members of the SWI/SNF nucleosome remodeling complex, namely BRM and BAF57, MeCP2 imparts transcriptional silencing in a DNA methylation-dependent fashion (Harikrishnan et al., 2005; Maxwell et al., 2013). MeCP2 recruitment of ATRX alters chromatin looping for several imprinted genes in the brain, which lose their imprinting status upon *Mecp2* depletion (Horike et al., 2005; Kernohan et al., 2010; Kernohan et al., 2014). Finally, MeCP2 is thought to promote heterochromatin condensate formation through competition between MeCP2 and linker histone H1 and selective recruitment of heterochromatic factors over euchromatic factors (Nan et al., 1997; Wang L. et al., 2020; Watson et al., 2026). Condensate expansion is counterbalanced by the MeCP2-LEDGF interaction (Lesire et al., 2025). However, the role of MeCP2 in condensate formation has drawn scrutiny from studies profiling condensate formation in non-murine models and interrogating the MeCP2-H1 interaction at the single-molecule level (Pantier et al., 2024; Chua et al., 2024). By partnering with diverse factors across the chromatin layers, MeCP2 expands the breadth of MeCP2’s molecular functionality and indicates how MeCP2 brings about transcriptional regulation across epigenetic layers. How MeCP2 shifts from a local to global chromatin regulation remains a major question in the field and likely requires future studies to comprehensively profile and integrate MeCP2 PTMs, co-factors, and multi-omics readouts to refine our understanding. Moreover, how MeCP2 contributes to higher-order chromatin organization in non-neuronal models is unknown but could corroborate and refine our understanding of MeCP2 in the chromatin landscape.

### 2.5 MeCP2-RNA interactions

The role of MeCP2 as an RNA-binding protein is newly appreciated in the field and much remains to be explored. MeCP2 has a short RG repeat motif that overlaps with its TRD and AT-hook motif and represents a noncanonical RBD (Thandapani et al., 2013). This motif is required and sufficient for RNA complex formation and displays heightened affinity for cytosine-rich dsRNA with large loops and multi-stem structures (Jeffery and Nakielny, 2004; Melikishvili et al., 2025; Good et al., 2026). MeCP2 displays a mutually exclusive preference for methylated DNA or RNA, which was validated *in vitro* when RNA-bound MeCP2 precluded interactions with NCoR1 (Jeffery and Nakielny, 2004; Good et al., 2026). Although the non-canonical extended AT-hook motif has high affinity for dsRNA, how this motif mediates MeCP2-RNA interactions remains to be tested (Filarsky et al., 2015). Looking more broadly, several reports confirmed that MeCP2 protein complexes are dependent on the presence of RNA which suggests MeCP2 may function as a multi-macromolecule signal integration hub in certain contexts (Young et al., 2005; Long et al., 2011). For investigators studying MeCP2-protein interactions, these findings may inform technical considerations. Altogether, these reports confirm that MeCP2 engages with RNA through multiple protein domains.

Subsequent studies have identified MeCP2-RNA co-factors and begun to understand their biological significance. Various RNA species including miRNAs, circRNAs, snoRNA, and lncRNAs are known to interact with and mediate MeCP2 localization and transcriptional and post-transcriptional regulation (Table 2; Figure 1). Using RNA immunoprecipitation sequencing (RIP-seq) in mouse primary cortical neurons, numerous miRNAs were identified. In the *Mecp2* null mouse setting, these miRNAs demonstrated significant target gene downregulation, suggesting that MeCP2-miRNA binding may indirectly upregulate miRNA target gene expression (Khan et al., 2017). When profiling differential circRNAs in mouse brain regions, Siqueira and colleagues found that MeCP2 binds to all circSIRT2 species. Their report details a potential mechanism of post-transcriptional regulation that could explain reduced SIRT2 protein levels when there is no change in SIRT2 mRNA or MeCP2 promoter occupancy (Siqueira et al., 2021). When bound to RNAs, MeCP2 displays altered DNA affinity and transcriptional targets as observed with circSCMH1 in neurons or *SNORD33* in breast cancer cells (Yang et al., 2020). The relationship between MeCP2 and lncRNAs is the most extensively studied given the high enrichment of lncRNAs, including *Rncr3*, from RIP-seq in the mouse cerebellum (Maxwell et al., 2013). More recently, it was demonstrated that MeCP2 binds to methylated *RNCR3* and suppresses the expression of the embedded miR-124a to coordinate neural differentiation (Zhang J. et al., 2024). Similarly, Mecp2 binds to the methylated *Mhrt* lncRNA to govern sex-specific transcriptional regulation in the mouse heart (K N et al., 2019). It was also observed that *HSATII* reactivation in various cancer and noncancer models sequesters MeCP2 into nuclear bodies and triggers genomic instability (Hall et al., 2017; Landers et al., 2021). Validation of the MeCP2-*NEAT1* complex *in vitro* represses the long *NEAT1* transcript isoform 2 (*NEAT1_2*) and requires the MeCP2 RBD (Cheng et al., 2018; Good et al., 2026). Although a direct interaction between *Evf2* and Mecp2 has not been confirmed, loss of *Evf2* in the embryonic mouse brain prevents Mecp2 occupancy and regulation of the *Dlx5/6* regulatory element (Bond et al., 2009). These interactions continue to exemplify how interplay between non-coding RNAs and MeCP2 exert contextual transcriptional regulation and showcase how MeCP2-governed RNA regulatory axes alter cell fates.

**TABLE 2:** MeCP2 RNA interactors.

Class	Interactor	Method	Model	Citation
miRNA	miR-375	RIP-Seq, qRT-PCR	Mouse primary cortical neurons and cerebellum	(Khan et al., 2017)
	miR-126	RIP-Seq, qRT-PCR	Mouse primary cortical neurons and cerebellum	(Khan et al., 2017)
	*Let-7i*	RIP-Seq, qRT-PCR	Mouse primary cortical neurons and cerebellum	(Khan et al., 2017)
lncRNA	*RNCR3*	RIP-Seq, qRT-PCR	Mouse cerebellum	(Maxwell et al., 2013)
	*NEAT1_2*	RIP, qRT-PCR	HEK293 cells	(Cheng et al., 2018)
		RIP, qRT-PCR	HEK293 cells	(Good et al., 2026)
	*MHRT*	RNA-ChIP	Mouse heart	(Harikrishnan et al., 2019)
	*HSATII*	RNA-IP, co-localization	Cancer cell lines	(Hall et al., 2017)
		Co-localization	HeLa and fibroblast cells	(Landers et al., 2021)
circRNA	circ945	RT-qPCR	ReNCell VM cells	(Siqueira et al., 2021)
	circ96	RT-qPCR	ReNCell VM cells	(Siqueira et al., 2021)
	circ948	RT-qPCR	ReNCell VM cells	(Siqueira et al., 2021)
	circ951	RT-qPCR	ReNCell VM cells	(Siqueira et al., 2021)
	circSCMH1	qPCR	Primary cortical neurons, HEK293T cells	(Yang et al., 2020)
snoRNA	*SNORD33*	RNA-pull down-MS, RIP-qPCR	MDA-MB-231 cells	(Wang B. et al., 2022)

## 3 MeCP2 in diseases and disorders

### 3.1 MeCP2 in neurodevelopmental and neuropsychiatric disorders

MeCP2 is essential for postnatal brain development and contributes to several neurodevelopmental and neuropsychiatric disorders. The best characterized disorder is Rett Syndrome (RTT), a rare progressive neurodevelopmental disorder affecting girls where 90–95% of cases present with *MECP2* pathogenic loss-of-function mutations (Amir et al., 1999; Liu et al., 2025b). Children with RTT achieve normal developmental milestones during the first 6–18 months before experiencing progressive developmental regression, loss of motor coordination, and autism-like symptoms. Beyond these neurological hallmarks, patients display multisystem pathophysiology with immune, microbial, cardiac, bone, endocrine, and metabolism imbalance (Liu et al., 2025a). Immune dysfunction is reviewed elsewhere (Figure 2). In summary, RTT patients display heightened systemic inflammatory response likely due to altered levels of pro-inflammatory mediators, disrupted IFN-γ and NF-κB signaling, reduced CD8^+^ T cells and natural killer (NK) cells, and elevated IgA antibodies(Pecorelli et al., 2020; Gonçalez et al., 2024). At the molecular level, RTT mutations disrupt MeCP2 co-factor binding, protein stability, PTMs, and transcriptional regulation (Lyst et al., 2013; Ebert et al., 2013; Yang et al., 2016; Sheikh et al., 2017; Tillotson and Bird, 2020). These perturbed epigenetic events have downstream consequences for neuron development and maturation and alter senescence, oxidative stress, ferroptosis, DNA damage, autophagy, and lipid metabolism in neurons and non-neuron models (Asaka et al., 2006; Ahmad et al., 2025). Although RTT patients are primarily diagnosed using neurological symptoms, RTT is now recognized as a multisystem disorder (Liu et al., 2025a).

**FIGURE 2:**
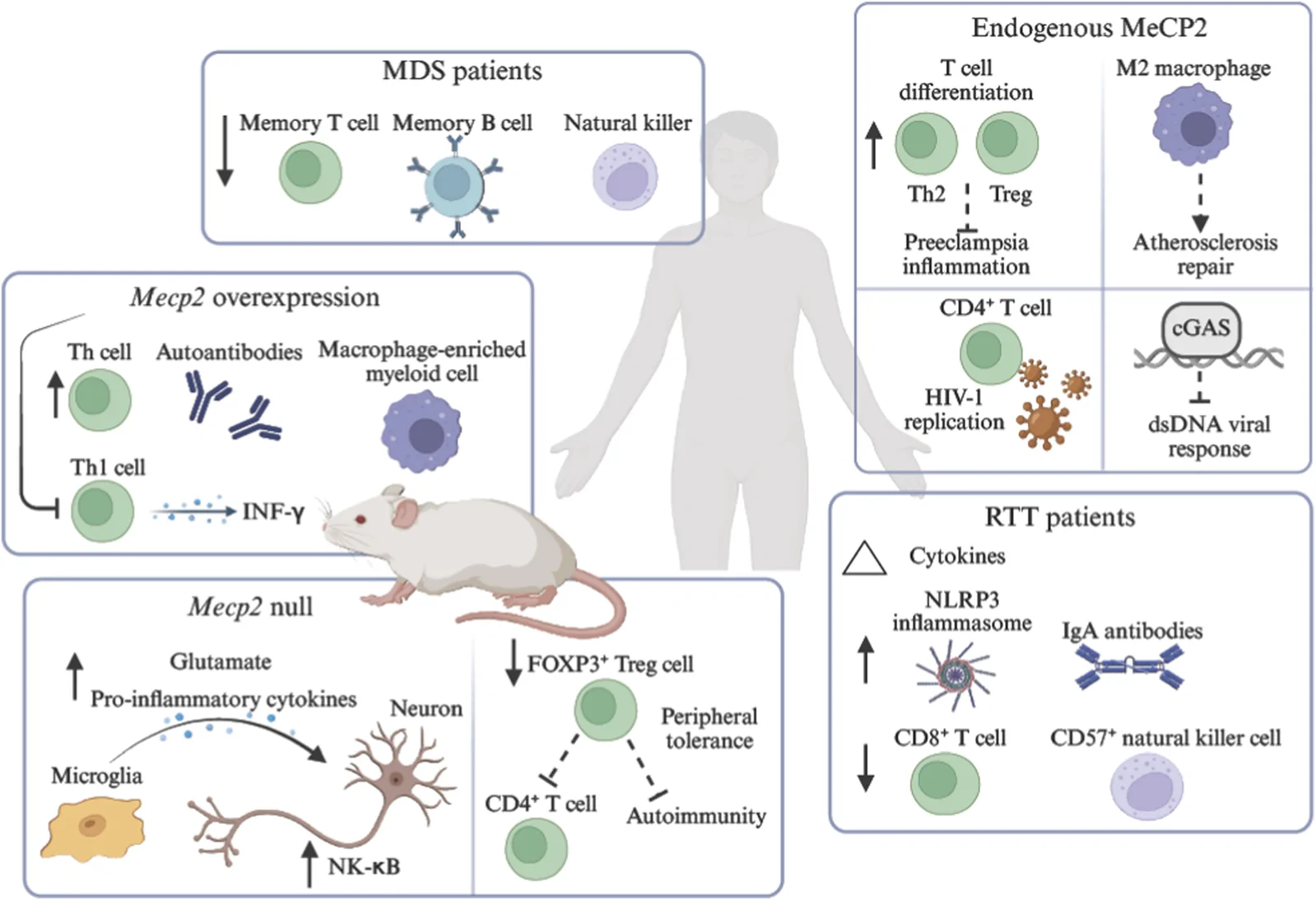
MeCP2 in immune homeostasis. Patients with *MECP2* loss-of-function (RTT) or amplification (MDS) present with altered inflammation and immune cell compositions. RTT patient have reduced cytotoxic CD8^+^ T cell and CD57^+^ natural killer cells along with elevated inflammatory markers (i.e., inflammasome activation, IgA, and cytokine profiles). Mice with T cell-specific *Mecp2* deletion gradually lose FOXP3 expression and cannot differentiate into FOXP3+ T regulatory (Treg) cells. Loss of this subset weakens peripheral tolerance allowing for spontaneous CD4^+^ T cell activity and enhanced autoimmunity features. In the central nervous system, *Mecp2* null mice present with increased neuroinflammation from microglia glutamate and pro-inflammatory cytokines release and heighted neuronal NF-κB activation. When MeCP2 expression is amplified on the other hand, patients have reduced peripheral memory T and B cells and natural killer cells. Mice models identified disfunction T helper 1 (Th1) response but overall elevated Th cells in addition to increased autoantibodies contributing to autoimmunity. In other settings, endogenous regulation of MeCP2 is linked with immune cell function and differentiation and anti-viral response. MeCP2 supports anti-inflammatory Th2 and Treg differentiation and cytokine release during preeclampsia and pro-repair M2 macrophage polarization upon MeCP2 K271 lactylation during atherosclerosis. MeCP2 regulates viruses in a context-dependent manner. In the setting of HIV-1, a single-strand RNA virus, MeCP2 loss is linked with enhanced viral replication in CD4^+^ T cells. However, for dsDNA viruses, cytosolic MeCP2 complete with cGAS for dsDNA thereby muting cGAS-mediate interferon response. Created in https://BioRender.com.

In *MECP2* duplication syndrome (MDS), duplications of the Xq28 locus that encodes *MECP2*, and typically *IRAK1*, results in intellectual disability, recurrent infections, central nervous system deterioration, and early death in males (Akaba and Takahashi, 2025). These symptoms may arise from increased susceptibility to autoimmunity diseases including systemic lupus erythematosus (Sawalha, 2013; Li Y. et al., 2024). At the molecular level, MDS cortical organoids display abnormal mTOR signaling and neuron morphology and activity. This is attributed to miR-199a-5p activity downstream of MeCP2 (Akaba et al., 2025). Figure 2 highlights immunological abnormalities observed in MDS patients including reduced memory T and B cells and natural killer cells. An MSD mouse model displayed compromised T helper type 1 (Th1)-mediated interferon-γ cytokine production while T helper cell type 2 (Th2) remained functional (Yang et al., 2012). When *Mecp2* is overexpressed in a systemic lupus erythematosus mouse model, researchers observe elevated helper T cells (Th) and autoantibodies in addition to activated macrophage-enriched myeloid cells (Li Y. et al., 2024). These reports demonstrate how *MECP2* amplification has implications in brain and immune cells.

Beyond RTT and MDS, *MECP2* is also linked to severe neonatal encephalopathy, autism spectrum disorder, Angelman-like syndrome, PPM-X syndrome, and X-linked intellectual disability (Watson et al., 2001; Couvert et al., 2001; Klauck et al., 2002; Carney et al., 2003). The best characterized, however, are genetic polymorphisms in the *MECP2/IRAK1* locus. These polymorphisms are associated with increased risk of developing autoimmune diseases marked by chronic inflammation such as systemic lupus erythematosus, primary Sjogren’s syndrome, and rheumatoid arthritis (Sawalha et al., 2008; Cobb et al., 2010; Han et al., 2013). Altogether, these MeCP2-driven pathologies underscore how MeCP2 function and dosage are critical for physiological homeostasis within and beyond the brain. Importantly, this summary highlights the need to pinpoint how MeCP2 directly orchestrates genetic and molecular pathways leading to cellular and immune dysregulation beyond correlative phenotypic studies.

### 3.2 MeCP2 in immune homeostasis

A major unknown in the field is to what extent and the mechanistic basis by which MeCP2 influences the tumor immune microenvironment. At present, there is a clear role for MeCP2 in the regulation of immune cell function and immune cell lineage specification. There is also a clear link between MeCP2 and cancer hallmarks, which will be elaborated upon in the next section. However, more investigation is needed to clearly establish the mechanistic bridge linking MeCP2 with cancer immunology. MeCP2 amplification in immune cells could have important implications for blood cancers, but more work is needed to clarify the role of MeCP2 in lymphomas and leukemias and how it impacts their epigenome. Ongoing efforts continue to clarify whether the role of MeCP2 in the context of neuroinflammation holds true in the tumor microenvironment (TME) and to what extent MeCP2 influences immune cell trafficking the TME (Cao et al., 2024). Whether the impact of MeCP2 on tumor-infiltrating lymphocytes or myeloid cells is largely due to its tumor-cell intrinsic or extrinsic effects needs to be clarified.

In the case of tumor-cell intrinsic regulation, the current literature suggests two potential mechanistic avenues whereby MeCP2 controls expression of immune cell surface receptors and secreted factors. To begin, studies reveal that MeCP2 regulates MHC expression on tumor cells. It is well established that MHC I expression impacts functionality of CD8^+^ T cells that infiltrate the TME. Loss or reduction of MHC I on tumor cells is a mechanism by which tumor cells evade adaptive immunity influences immunotherapy response (Sade-Feldman et al., 2017). Interestingly, MHC class I genes are particularly rich in CpG islands. Studies show ectopic expression of MeCP2 downregulates cell-surface expression of MHC class I molecules in neuroblastoma cells in response to interferon (Miralvès et al., 2007). These data suggest that high levels of MeCP2 may contribute to the repression of MHC expression, but more investigation is needed to determine the physiological cellular context where this occurs and if MeCP2 expression status is clinically relevant in immunotherapy response.

MeCP2 is implicated in the epigenetic silencing of TME-modulating extracellular proteins ranging from chemokines, cytokines, and extracellular matrix factors. MeCP2 expression is amplified across cancers thereby increasing the potential for secreted factors dysregulation (Neupane et al., 2016). Examples of these factors include secreted frizzled related proteins (SFRPs), Dickkopf (DKK), and Wnt inhibitory factors (WIFs) which target different components of the receptor-ligand complex critical for Wnt signaling and are epigenetically silenced during tumor progression (Suzuki et al., 2008). Because immune cells express receptors activated or inhibited by these factors, understanding the extent by which MeCP2 regulates tumor-intrinsic immunomodulation is an important direction for future studies. SFRPs are the best characterized and their promoters have been shown to frequently undergo CpG hypermethylation and silencing in cell lines, xenografts and primary colon tumors (Suzuki et al., 2004). SFRP4 expression correlates with immune cell infiltration of CD8^+^ T cells and checkpoint proteins in the context of gastric cancer (Yu et al., 2022). However, this report was a correlative study, and the mechanistic link remains to be determined. Direct evidence connects MeCP2 overexpression with SFRP4 inhibition in cardiomyocyte hypoxia/reperfusion and rheumatoid arthritis models (Miao et al., 2013; Li et al., 2021). Since Rheumatoid arthritis is an autoimmune disease where cytokine imbalance and altered Wnt pathway activation participates in the pathogenesis, this same principle could be at work in the TME, although direct testing across different cancer models is still needed. Promoter methylation and silencing of DKK and WIF are also linked with MeCP2. Inhibition of MeCP2 in osteoblasts leads to increased expression of DKK1 and altered osteoblast differentiation, thereby establishing a functional MeCP2-DKK1 signaling axis (Dong et al., 2026). Moreover, mechanistic studies reveal that MeCP2 inhibits WIF1 and contributes to TGF-β1-induced fibrosis (Sha et al., 2023). MeCP2 is also linked with TGF-β signaling in endothelial cells and augments endothelial-to-mesenchymal transition. This occurs through MeCP2 suppression of BMP7 by directly binding its promoter and increasing promoter CpG island methylation (Li Z. et al., 2019). This report is consistent with the paradigm whereby MeCP2 silences TME-modulating secreted factors since BMP7 regulates tumor progression and ratios of immune cell infiltration of the TME (Katsuta et al., 2019). Another secreted factor, IL6, is subject to MeCP2-induced silencing in pancreatic adenocarcinoma cell lines (Dandrea et al., 2009). The presented studies suggest tumor-intrinsic MeCP2 may be involved in modulating immune cell TME infiltration and activity. However, this remains an important question that has yet to be addressed to any significant depth with the appropriate models to elucidate mechanistic evidence. It is also conceivable that this paradigm could extend beyond extracellular secreted factors and immune infiltration.

MeCP2 also has the potential to influence tumor-cell extrinsic properties by influencing the functionality and differentiation of specific immune cell subsets as observed in MDS. With respect to T cells, MeCP2 is known to directly regulate T cell differentiation and viral response. MeCP2 is critical for differentiation of naïve CD4^+^ T cells into Th1 and Th17 lineages. To establish mechanism, loss of MeCP2 in CD4^+^ T cells was shown to impair the expression of miR-124. Since miR-124 was critical for repression of SOCS5, its increase led to inhibition of STAT1 and STAT3, which are necessary for the differentiation of Th1 and Th17 cells, respectively (Jiang et al., 2014). The overexpression of MeCP2 was shown to dysregulate anti-inflammatory Th2/Treg cells and cytokine release through downregulation of SFRP4 (Peng et al., 2025). Since these studies were performed in the context of preeclampsia, it remains unclear how MeCP2-mediated control of placental inflammation and T cell homeostasis via SFRP4 bioavailability extends to the TME across different solid tumors. Additionally, in CD4^+^ T cells infected with human immunodeficiency virus type I (HIV-1), miR-422a suppresses MeCP2 and genetic ablation of MeCP2 promotes HIV-1 replication. The authors confirm depletion of miR-422a activates interferon-stimulated genes and inhibited HIV-1 replication. It remains inconclusive if MeCP2 hinders the HIV-1 lifecycle by directly or indirectly activating CD4^+^ T cell interferon and inflammation response (Du et al., 2026). Another report shows MeCP2 function directly in the spleen. This study used spleen-targeting lipid nanoparticles encapsulated with antisense oligonucleotides targeting Mecp2. Splenic immune cells analysis revealed that concomitant with a reduction in splenic *Mecp2* levels, there was a reduction in splenic B and T cells, and a reduction in the levels of autoantibodies for nuclear antigens. Reduced expression of immunomodulating IL-5, TNF-α, CCL20 and TGF-β1 was also observed (Zhang S. et al., 2025). Interestingly, each of these cytokines and secreted factors have been shown to be important in dysfunctional immune cell infiltration of the TME.

With respect to macrophages, MeCP2 can regulate tumor-associated macrophage (TAM) development and macrophage differentiation. Integration of single-cell multi-omics datasets identified that PPARG, NFAT5 and MeCP2 promote the pro-tumorigenic SPP1^+^ TAM lineage from peripheral monocytes. Conversely, MAF, HES1 and PRDM1 promote the C1QC^+^ TAM linage from tissue-resident macrophages. These results implicate MeCP2 as a pivotal regulator of pro-tumorigenic TAMs but more work is needed to mechanistically validate this development pathway and its relevance in the TME (Xiong et al., 2026). In the context of atherosclerosis, MeCP2 undergoes exercise-induced K271 lactylation in macrophages. This PTM promotes anti-inflammatory M2 macrophage polarization to ameliorate atherosclerosis pathologic features. At the epigenetic level, H3K36me3 interacts with lactylated MeCP2 to increase chromatin accessibility and repress RUNX1 transcription factor expression (Chen et al., 2024). This report establishes a clear molecular link by which MeCP2 protein co-factors give rise to chromatin organization and dictate macrophage differentiation.

### 3.3 MeCP2 in cancer

Altered DNA methylation patterns have been widely associated with carcinogenesis (Baylin and Jones, 2011). As a DNA methylation reader, MeCP2 is increasingly linked to clinicopathological, prognosis, therapy response, and molecular adaptations in numerous cancer types. A pan-cancer bioinformatics study using publicly available cancer databases reported that *MeCP2* expression correlates with clinical outcomes and associates with immune cell microenvironment infiltration (Wang Y. et al., 2022). However, MeCP2 alterations and correlations with tumor mutation burden and microsatellite instability are unique to each cancer type. In contrast to MeCP2 neurological pathologies, around 4% of patients within each cancer type undergo *MECP2* amplification, mutation, and deep deletion alterations. Along these lines, an unbiased screen of nonmutated genes identified MeCP2 as an oncogenic gene when overexpressed (Neupane et al., 2016). The authors found that *MeCP2* is amplified in ∼18% of cancers, and *MECP2* amplification and *RAS* family oncogene alterations are mutually exclusive for a subset of cancers. This clinical observation is supported by MeCP2-induced MAPK and PI3K pathway activation, which are frequently hyperactivated in RAS-driven cancers. Accumulating clinical, diagnostic, molecular, and genetic evidence across *in vitro* and *in vivo* cancer models have begun to delineate the contextual role of MeCP2 in this disease as described in previous reviews (Mahmood and Rabbani, 2019; Nejati-Koshki et al., 2023). In the following sections, we will discuss the latest functions of MeCP2 in various cancer settings.

#### 3.3.1 Breast cancer

Breast cancer remains the most prominent cancer type diagnosed in women, accounting for 32% of cases diagnosed and 15% of cancer-related deaths (Siegel et al., 2024). MeCP2 is recognized as a putative oncogene in this cancer type (Nejati-Koshki et al., 2023). Early clinical reports exploring the correlation between MeCP2 expression status and clinical outcomes arrived at disparate results (Billard et al., 2002; Müller et al., 2003). These conflicting conclusions could be the result of non-neoplastic tumor contamination or differing sample quality or histology. Immunohistochemical analysis later confirmed that both MeCP2 and DNMT1 are significantly elevated in invasive ductal carcinoma compared to non-neoplastic lesions (Xu et al., 2012). More recently, The Cancer Genome Atlas (TCGA) identified MeCP2 as a prognostic telomere maintenance-related gene and confirmed an association between increased transcript levels and poor prognosis (Huang et al., 2025). Also of note, *MeCP2* is elevated during mammary gland formation and acinar cell differentiation indicating that expression may be developmentally regulated and reactivated in carcinomas (Billard et al., 2002; Plachot and Lelièvre, 2004).

Numerous studies using breast carcinoma cell models detail how MeCP2 drives breast cancer transcription at methylated promoters. The link between MeCP2 and ERα is the best studied, likely due to the fact the ∼70% of breast cancers are ER-positive (i.e. luminal A and B). MeCP2 is associated with the ERα minimal promoter element and epigenetically controls *ESR1* and its target gene expression when bound to methylated promoters in conjunction with DNMT1, HDAC1, and mutant p53 (Reid et al., 2005; Rasti et al., 2012; Zhu et al., 2012; Arabsolghar et al., 2013). ERα expression can be induced in ER-negative cells using DNMT and class I/II histone deacetylase (HDAC) inhibitors resulting in promoter hypomethylation, loss of MeCP2 and MBDs binding, and a shift towards chromatin accessibility via histone PTMs (Sharma et al., 2005). Altogether, these studies identify MeCP2 as a negative ERα transcriptional regulator, and further investigation could determine if MeCP2 expression informs hormone therapy response. Beyond *ESR1*, MeCP2 binds to hypermethylated promoters and transcriptionally silences genes involved in tumor-associated antigens (i.e., *MAGE-A*), cell polarity (i.e., *CLDN6*), tumorigenesis (i.e., *CD44*, *PTEN*, *CCND2*, *ADAM12*, *HIF1A*), and tumor suppression (i.e., *BRCA1*, *GSTP1*, *RASSF1*, *RARB2*, *PTEN*, *KLK6*) genes (Magdinier et al., 2000; Pampalakis et al., 2009; Müller et al., 2010; Xu et al., 2012; Ray et al., 2013; Li C. et al., 2019). Chromatin immunoprecipitation sequencing (ChIP-Seq) identified MeCP2 loci enriched for repetitive and retrotransposon elements, suggesting that MeCP2 may in turn regulate genome stability at these loci (Ballestar et al., 2003; Koch and Strätling, 2004). MeCP2 has been linked to broadly dysregulated transcription in breast cancer. Castro-Piedras and colleagues were the first to use RNA-Seq in luminal A and TNBC cell models and confirmed MeCP2 transcriptional activation and suppression of various genes, although further validation is required (Castro-Piedras et al., 2020). These examples showcase the ability of MeCP2 to modulate the breast cancer transcriptome.

As a transcriptional regulator, MeCP2 dictates various breast cancer hallmarks including proliferation, motility, and survival during cytotoxic and apoptotic stress (Figure 3). MeCP2 can enhance proliferation in various breast cancer subtypes and BALB/c nude mice. Tong and colleagues found that MeCP2 localizes to the methylated *RPL5* and *RPL11* promoters and downregulates protein expression. This enabled MDM2-mediated p53 ubiquitination and degradation, subsequently enhancing cell proliferation (Tong et al., 2020a). Numerous reports implicated MeCP2 as a cell adhesion and motility enhancer. Notably, exogenous MeCP2 e2 expression in primary human mammary and breast epithelial cells conferred anchorage-independent growth and MAPK pathway activation comparable to activated HRAS^V12^. This phenotype was specific to the e2 isoform and required an intact MBD (Neupane et al., 2016). Subsequent studies report conflicting *in vitro* invasion and migration results in luminal A models (Tong et al., 2020b; Jiang et al., 2020). Many publications support a role for MeCP2 in cell motility and epithelial-mesenchymal-transition (EMT). MeCP2 can promote lung metastases through regulation of FOXA1 adhesion target genes (Liu et al., 2026). Other studies observe elevated EMT and invasion when MeCP2 transcriptionally silences the *KLK6* or *CLDN6* promoters (Pampalakis et al., 2009; Xu et al., 2012). Most recently, MeCP2 was linked to EMT under hypoxic conditions through splicing regulation. PRMT5 recruited DNMT3A and MeCP2 to an alternative-spliced *TCF3* intronic locus where MeCP2 led to RNA pol II pausing and an EMT-promoting splice isoform (Mutnuru et al., 2025). MeCP2 is also linked to antineoplastic resistance. By transcriptionally silencing the methylated *MDR1* promoter along with MBD2, MeCP2 may offer a cellular pathway to achieve *MDR1-*mediated drug resistance (David et al., 2004). As mentioned in the RNA interactor section, *SNORD33*-bound MeCP2 decreases MeCP2-target gene binding. The authors suggest that this interaction alters gene expression, thereby contributing to cell survival in platinum resistant cell models (Wang B. et al., 2022). Critical translational and post-translational regulation of MeCP2 in breast cancer is missing in the literature and may unravel contextual MeCP2 biology. MiR-194-3p and miR-106b-5p target MeCP2 for degradation resulting in suppressed or enhanced oncogenic cell properties, respectively (Zhou et al., 2020; Xue et al., 2025). Additionally, the functional significance of MeCP2 acetylation PTMs and CXXC5, the only protein co-factor identified in breast cancer, remains unknown (Castro-Piedras et al., 2020; Ayaz et al., 2021). Future studies are required to comprehensively map MeCP2 co-factor interactions and integrate unbiased transcriptome and chromatin accessibility datasets to deepen our understanding of breast cancer.

**FIGURE 3:**
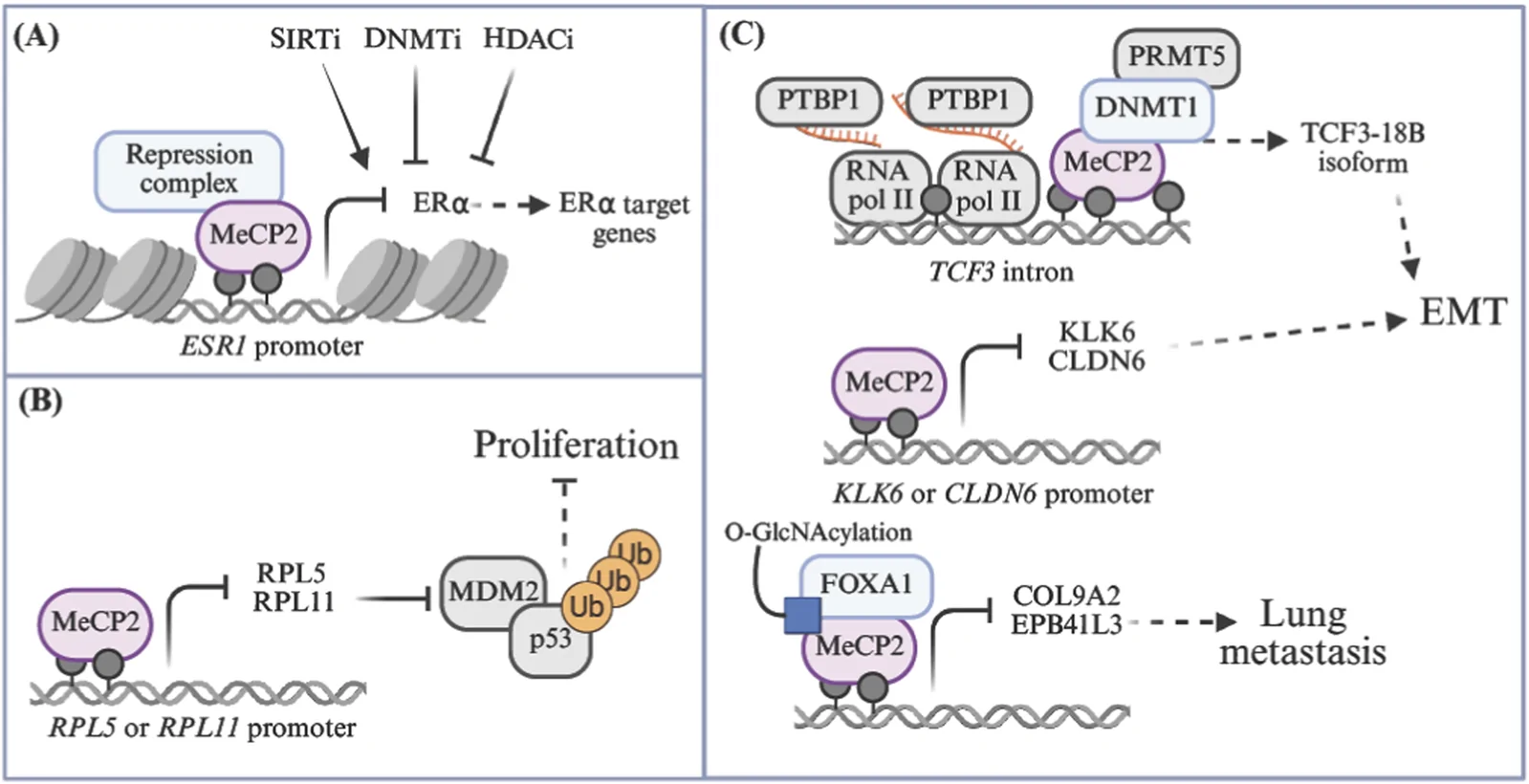
Molecular pathways of MeCP2 in breast cancer. **(A)** MeCP2 negatively regulates the expression of ERα, a key driver of ER-positive tumors, through its recruitment of a repression complex (i.e., DNMT1, HDAC1, and mutant p53, MDB2) to the methylated *ESR1* promoter leading to suppressive DNA methylation and histone marks. Inhibiting the acetylase activity of Sirtuins enforces ERα suppression while DNMT and HDAC inhibitors restore ERα expression. **(B)** MeCP2 suppresses RPL5/11 expression when bound to their methylated promoters. This releases MDM2 negative regulation and allows MDM2 to target p53 for ubiquitination-mediated degradation and promote cell proliferation and survival. **(C)** MeCP2 engages several pathways to directly or indirectly promote cell motility and EMT. During hypoxic conditions, MeCP2 occupancy at a methylated *TCF3* intron, due to PRMT5-directed DNMT1 activity, leads to RNA pol II pausing. This allows time for PTBP1 to alternatively splice the EMT-promoting TCF3-18B isoform. Alternatively, MeCP2 occupancy at the methylated regions shuts down transcription of *KLK6*, *CLD6*, and O-GlcNAcylated-FOXA1 adhesion-related genes. This transcriptional tuning enhances EMT-like phenotypes and lung metastasis *in vivo*. Created in https://BioRender.com.

#### 3.3.2 Colorectal cancer

Colorectal cancer (CRC) is the third most common cancer diagnosed and second leading cause of cancer-related death in the world, with rising rates of diagnosis (Eng et al., 2024). Clinical evidence supports a pathological role for MeCP2 in CRC. Bioinformatics analyses of CRC patients associate high *MECP2* expression with poor prognosis, worse overall survival, and a highly stromal tumor microenvironment (Wang Y. et al., 2022). Additionally, MeCP2 expression is elevated at both the RNA and protein levels in patients with metastatic CRC compared to those with non-metastatic disease, suggesting a strong association between MeCP2 expression and metastatic progression (Zhou and Guo, 2018; Luo and Ge, 2020). Patients with chronic inflammatory conditions, such as ulcerative colitis (UC) and inflammatory bowel disease, are predisposed to develop CRC (Eng et al., 2024). Interestingly, a research group reported that two out of ten patients with UC-induced CRC had an in-frame mutation of MeCP2 (Yan et al., 2019). This patient evidence links MeCP2 expression with CRC, but significantly more work is needed to establish the role of MeCP2 in colitis-associated cancer development.

Functional studies support these clinical observations and suggest a role for MeCP2 as a driver of CRC progression. Several mechanisms have been proposed to explain the pro-tumorigenic effects of MeCP2. A recurring theme is the ability for MeCP2 to silence tumor suppressor genes through epigenetic repression. For example, reactivation of the hypermethylated *MLH1* and *PDLIM4* tumor suppressor genes is observed following DNMT inhibition and is associated with reduced MeCP2 binding at these sites. The combination of this hypomethylating agent and platinum-based chemotherapy agents produced synergistic tumor-killing *in vitro* (Qin et al., 2015). Similarly, treatment with cardiac glycosides compounds could restore the expression of genes normally silenced in CRC cells. These compounds activate calcium-calmodulin kinase (CamK), which blocks MeCP2 nuclear localization and prevents MeCP2-mediated repression of tumor suppressor genes, ultimately leading to CRC cell death (Raynal et al., 2016). Another study demonstrated that proper MeCP2 recruitment of repressor factors, such as HDAC1 and ATRX, depends on MeCP2 K171 deacetylation status. Disrupting these interactions through K171 acetylation significantly impaired CRC cell growth and connects the MeCP2 repressive complex with CRC progression (Pandey et al., 2015). Unbiased profiling of MeCP2 genome occupancy and transcriptional regulation has yet to be performed and is a major knowledge gap for this cancer type. Collectively, these findings suggest that the oncogenic functions of MeCP2 depend, at least in part, on its ability to establish and maintain transcriptional repression at tumor suppressor and/or anti-proliferative gene loci.

MeCP2 contributes to multiple colorectal cancer hallmarks including proliferation, EMT, and chemotherapy resistance (Figure 4). Genetic depletion of *MECP2* in CRC cell models confirmed that MeCP2 enhances cell growth in 2D and 3D settings (Song et al., 2016; Luo and Ge, 2020). MeCP2 also promotes metastasis through regulation of several pathways linked to EMT and invasive behavior. A computational analysis approach associated elevated MeCP2 expression with metastatic disease in patients (Zhou and Guo, 2018). This observation was confirmed through *in vitro* and *in vivo* models where MeCP2 promotes cell migration, invasion, and metastatic outgrowths (Song et al., 2016; Luo and Ge, 2020; Wang et al., 2021). Mechanistically, MeCP2 recruits the SPI1 transcription factor to the *ZEB1* promoter to induce expression of this key mesenchymal regulator. This regulation may contribute to the pronounced degree of lung metastatic nodules *in vivo* (Luo and Ge, 2020). Moreover, MeCP2 is linked with downregulation of E-Cadherin, a key epithelial lineage and cell-cell adhesion protein frequently lost during tumor invasion and metastasis. Patient colorectal tissues exhibit increased promoter DNA methylation at the *CDH1* locus, accompanied by heterogeneous E-Cadherin expression that is inversely correlated with MeCP2 levels (Darwanto et al., 2003). These findings suggest that MeCP2 contributes to the acquisition of a more invasive phenotype by promoting a mesenchymal CRC transcriptional profile. MeCP2 also promotes metastatic behavior by silencing miR-137 to indirectly increase expression of the oncogene c-Met (Chen et al., 2017). Additionally, MeCP2 reduces *KLF14* mRNA N^6^-methyladenosine modifications and stability through its interaction with methyltransferase-like 14 (METTL14). As a result, decreased abundance of the *KLF14* tumor suppressor gene may facilitate metastasis (Wang et al., 2021). In addition to its roles in tumor growth and metastasis, MeCP2 may also contribute to chemotherapy resistance in CRC. MeCP2 can suppress the alternative splicing of osteopontin isoforms (OPNb/c) which promote 5-fluorouracil (5-FU) standard of care chemotherapy resistance. However, upon MeCP2 S421 phosphorylation or treatment with 5-FU, MeCP2 could no longer bind the OPN exons and inhibit OPN splicing (Chang et al., 2020). This finding expands the functional repertoire of MeCP2 beyond transcriptional repression in CRC and suggests that RNA processing may represent an additional mechanism through which MeCP2 promotes colorectal cancer progression. Further studies, particularly co-factor binding and *in vivo* models, are required to fully understand the role of MeCP2 in distinct CRC etiologies beyond transcriptional regulation.

**FIGURE 4:**
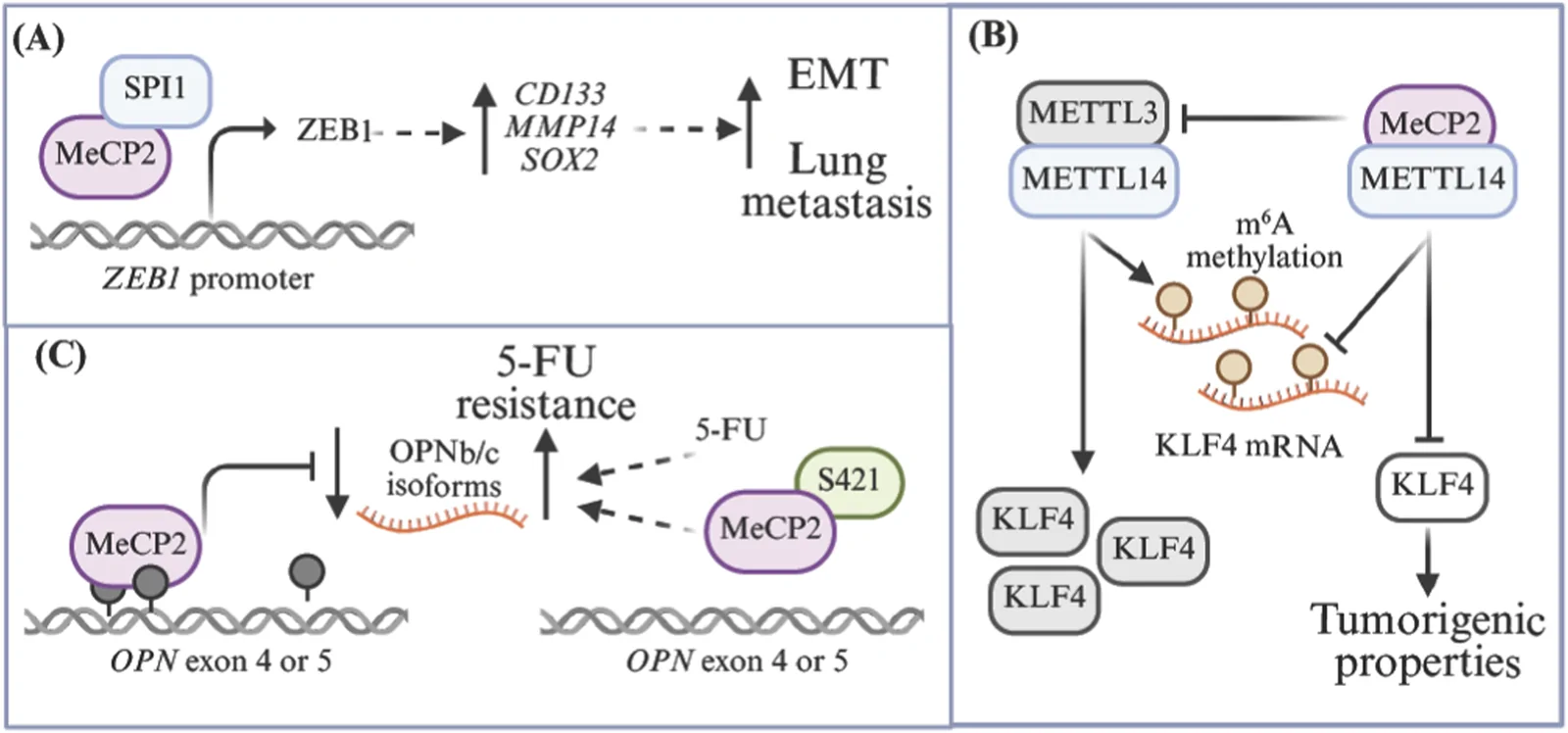
Molecular pathways of MeCP2 in colorectal cancer. **(A)** MeCP2 leads to the transcriptional activation of ZEB1 by recruiting the SPI1 transcription factor to its promoter. ZEB1 can activate expression of its target genes (i.e., CD133, MMP14, and SOX2) shifting CRC cells to a mesenchymal state and contribute to elevated *in vivo* lung metastatic nodules. **(B)** Another avenue MeCP2 utilizes to enhance CRC tumorigenic properties is through suppression of N^6^-methyladenosine mRNA modifications. It achieves this by sequestering METTL14 from METTL3 and suppresses its ability to deposit the N^6^-methyladenosine mark on *KLF* mRNA. This leads to *KLF* mRNA instability and reduced protein levels of this tumor suppressor. **(C)** Phosphorylation of MeCP2 S421 or loss of DNA methylation at the *OPN* exons contributes to 5-fluorouracil (5-FU) chemotherapy resistance. In these conditions, or in the presence of 5-FU, the authors observe loss of MeCP2-mediated *OPN* alterative splicing regulation and increase of the OPNb and OPNc isoforms which contribute to 5-FU resistance. Created in https://BioRender.com.

#### 3.3.3 Gastric cancer

Gastric cancer, also known as stomach cancer, is the sixth most common and seventh leading cause of cancer-related deaths worldwide with early-onset sporadic cases rising in females (Ben-Aharon et al., 2023). Analyses of MeCP2 expression in clinical gastric samples indicate conflicting clinical significance. Studies reported that *MECP2* abundance did not associate with any clinicopathological features and was not altered between matched carcinoma and non-carcinoma tissues (Fang et al., 2004; Wada et al., 2010). However, use of extended biospecimens collection and the TCGA database found that gastric carcinomas displayed elevated MeCP2 transcript and protein levels and expression correlated with tumor stage, poor differentiation, lymph node metastasis status, and poor overall and disease-free survival (Zhang J. et al., 2017; Zhao L. et al., 2017). Together, these comprehensive clinical analyses establish a potential clinically relevant role for MeCP2 in gastric carcinomas.

MeCP2 is a well-documented non-coding RNA transcriptional regulator in gastric cancer. To understand MeCP2 function globally, Liu and colleagues integrated ChIP-Seq, microarray, and DNA methylation datasets. They found MeCP2 localized to over 5,000 regions primarily in intergenic and intronic regions that overlapped methylated and unmethylated transcriptional start sites. Additionally, MeCP2 may play a broad role in gastric cancer biology as it positively and negatively regulated ∼1,300 genes (Liu Y. et al., 2020). Several reports confirm MeCP2 downregulates miRNAs and lncRNAs including miR-19a/b, miR-22, miR-122, miR-338-5p, miR-338-3p, GPC5-AS1, and linc00086 (Wada et al., 2010; Tong et al., 2016; Yang et al., 2018; Zhu et al., 2018; Tong et al., 2020a; Bo et al., 2022). MeCP2 may contribute to tumor growth by targeting miR-22. Re-expression of this miRNA resulted in *MECP2*, *MTHFD2,* and *MTHFR* degradation and decreased methyl donor SAM abundance. This led to transcription of the hypomethylated *p16*, *PTEN*, and *RASSF1A* tumor suppressor genes and subsequent cell cycle arrest and apoptosis (Tong et al., 2020b). In addition, MeCP2 can indirectly downregulate the proliferation inhibitors, p16 and p21, by silencing miR-338-5p and miR-338-3p at the methylated miR-338 promoter resulting in upregulation of the miRNA target genes (Tong et al., 2016).

MeCP2 is also an important transcriptional regulator responsible for enhanced proliferation, anti-apoptotic response, migration, chemotherapy resistance, and other pathways (Kim et al., 2016; Qin et al., 2017). MeCP2 contributes to *in vitro* and *in vivo* gastric cancer proliferation by regulating the methylated *FOXF1* and *MYOD1* promoters to enhance FOXF1/Wnt5a/β-Catenin signaling or inhibit MYOD1-mediated Caspase-3 apoptosis, respectively (Zhao L. et al., 2017). Regarding cell adhesion, MeCP2 may contribute to *ILK* silencing in an adhesion-mediated setting. The authors found that MeCP2 interacts with HDAC3 only in adhered cells where HDAC3 and H3K9me2 bind to the silenced *ILK* promoter. The authors postulate that MeCP2, HDAC3, and H3K9me2 function as an adhesion-mediated *ILK* repressive complex (Kim et al., 2007). Additionally, MeCP2 suppresses the methylated *FBXW7* promoter contributing to Notch1/c-Myc/mTOR pathway signaling, migration, and invasion (Zhao L. et al., 2022). Lastly, several publications demonstrate that MeCP2 is upregulated in 5-FU and cisplatin-resistant gastric cancer cell models and contributes to drug insensitivity. Mechanistically, MeCP2 can enhance NOX4 transcription and PKM2-mediated 5-FU resistance or downregulate PDK1 for enhanced proliferation, anti-apoptotic signals, and cisplatin-resistance (Qin et al., 2022; Guo et al., 2022). Interestingly, miR-19a/b is linked with multidrug resistance and engages in a negative regulatory feedback loop with MeCP2 (Zhu et al., 2018). These results indicate that a multi-pronged approach will be required to address drug resistance in gastric cancer. Additional MeCP2 post-transcriptional regulators in gastric cancer include miR-181c, miR-212, miR-340, miR-638, and miR-1324 (Wada et al., 2010; Hashimoto et al., 2013; Zhao L. Y. et al., 2017; Zhang et al., 2020). Importantly, re-expression of miR-181c, miR-340, or miR-638 decreases gastric cell proliferation and suppresses the MEK/ERK pathway (Hashimoto et al., 2013; Zhao L. Y. et al., 2017). Although no additional insights have been published since 2023, current evidence demonstrates a biologically significant role of MeCP2 in gastric cancer progression that could be further supported by identifying binding partners and how it contributes to chromatin architecture and splicing regulation.

#### 3.3.4 Pancreatic cancer

As of 2024, pancreatic ductal adenocarcinoma (hereafter referred to as pancreatic cancer) is the third leading cause of cancer deaths and displays one of the poorest 5-year cancer survival rates in the United States (Stoop et al., 2025). The field is beginning to delineate the relationship between MeCP2 expression and function in pancreatic cancer. Profiling clinical specimens through the Badea Pancreas and TCGA databases identified elevated MeCP2 transcript and protein abundance in tumor tissues that correlates with cancer stage (Wang H. et al., 2020). How MeCP2 expression associates with patient overall survival and other clinical features remains to be determined. Currently, several papers have connected MeCP2 to pancreas α/β cell identity, differentiation, and insulin regulation (Kuroda et al., 2009; Dhawan et al., 2011). These findings may be pertinent as diabetes is a known risk factor for pancreatic cancer (Stoop et al., 2025).

The use of *in vitro* and *in vivo* models has linked MeCP2 to transcriptional downregulation and oncogene-like properties. It was shown that MeCP2, along with H3K9me, can silence the methylated *IL-6* promoter in select pancreatic cancer cell models. MeCP2 occupancy negatively correlated with RNA polymerase II occupancy indicating that a mutually exclusive relationship may occur at this locus (Dandrea et al., 2009). A subsequent study discovered that *LIN28A*, an RNA-binding protein with tumor promoting properties, is re-expressed upon DNMT inhibition. Recruitment of MeCP2, but not MBD3, to the methylated LIN28A promoter was sufficient to silence transcription. LIN28A re-expression resulted in heightened stem cell gene expression, 2D and 3D proliferation, and invasive phenotypes, suggesting that MeCP2 plays a tumor suppressive function (Xu et al., 2016). Finally, Wang and colleagues connected MeCP2 to TGF-β and epithelial–mesenchymal signaling. *In vitro* and *in vivo* experiments indicate that MeCP2 contributes to pancreatic cancer proliferation, migration, and invasion. Molecularly, MeCP2 promoted matrix remodeling pro-MMP2/9 species and mesenchymal markers while simultaneously suppressing epithelial markers. Moreover, the MeCP2-SMAD2/3/4 protein complex contributes to *FURIN* upregulated to enhance FURIN-mediated TGF-β1 activation and SMAD2/3 phosphorylation (Wang H. et al., 2020). Although MeCP2 does not directly bind to the *FURIN* promoter, it may recruit histone or chromatin factors to induce *FURIN* epigenetic regulation. Overall, the role of MeCP2 in pancreatic cancer remains understudied and no additional insights have been published since 2023. Although the field has identified several functions of MeCP2 in pancreas biology, further investigation into MeCP2’s clinicopathological features, translational and post-translational regulation, co-factor identification, and broad epigenome modulation could be pursued.

#### 3.3.5 Hepatocellular carcinoma

Hepatocellular carcinoma (HCC) accounts for ∼90% of liver cancer cases with hepatitis B virus (HBV) infection and cirrhosis following hepatitis C virus infection are leading HCC risk factors (Llovet et al., 2021). The clinical significance of MeCP2 in HCC is mixed, but current evidence points to dysregulated MeCP2 expression and processes. One report detected no significant differences in *MEPC2* levels in cancerous tissue even though carcinomas displayed aberrant methylation and expression of DNA methylation writers (Saito et al., 2001). In an expanded clinical study of HCC, cirrhosis, and HBV infected patients, *MECP2* overexpression occurred in cirrhosis and carcinoma compared to non-cirrhosis tissue and correlated with liver cirrhosis and tumor staging (Li Y. et al., 2018). Interestingly, MeCP2 is linked with liver fibrosis through miR-214 (Zhao et al., 2025). Finally, in a genome-wide association study, inositol and phosphatidylinositol metabolism, glycerophospholipid catabolism, and MeCP2 were negatively associated with HCC risk (Hoang et al., 2025). These results provide an interesting connection between liver metabolic dysfunction seen in RTT syndrome and MeCP2’s contribution towards liver cancer (Liu et al., 2025a).

Mechanistically, MeCP2 enhances HCC proliferation, developmental signaling pathways, and HCC standard of care resistance. Investigators found that MECP2, MBD2, c-Ski, and DNMT3A regulators silence TTP independent of TGF-β1 stimulation. TTP repression elevated the transcript half-life of c-Myc and inhibited TGF-β1 antiproliferative signaling. Importantly, the relationship between TTP, c-Myc, and DNA methylation significantly correlated in clinical specimens, and indirectly implicate MeCP2 with c-Myc oncogene activity (Sohn et al., 2010). Another study found that MeCP2 drives cell proliferation through the upregulation of Cyclin D1, CDK4, and hedgehog signaling through the downregulation of PTCH1 (Wu et al., 2015). Zhao and colleagues showed that MeCP2 knockdown broadly leads to cell cycle arrest, apoptosis, and p38 pathway activation (Zhao et al., 2013). Recently, MeCP2 was linked with *FGGY* gene regulation via CoREST recruitment. This transcript regulation may modulate *FGGY* isoform expression to alter the HCC immune microenvironment (Zhao and Andersen, 2026). Finally, when studying the role of circRNAs in sorafenib resistance, Weng and colleagues observed elevated circFOXM1 abundance in resistant tissue that sponged miR-1324 and promoted cell growth upon sorafenib treatment. Although miR-1324 targets *MECP2*, future studies could directly explore the role of MeCP2 in sorafenib resistance (Weng et al., 2021). Overall, current evidence supports a tumor supportive role for MeCP2 which could be expanded upon using *in vivo* models, profiling of MeCP2’s post-translational and co-factor regulation and broad transcriptome and chromatin events.

#### 3.3.6 Prostate cancer

Prostate cancer remains the second leading cause of cancer deaths and accounted for 30% of all new male cancer diagnoses in 2024 (Siegel et al., 2024). The clinical significance of MeCP2 is poorly characterized, although cellular models have established a role for MeCP2 in prostate cancer. A comprehensive assessment of MBD family members identified MeCP2 overexpression in carcinoma cell lines, but not in patient tissues (Patra et al., 2003). Future studies could verify these results in alternative public databases or an ad-hoc clinical analysis to delve deeper into clinical features. Current *in vitro* studies link MeCP2 with androgen-independent proliferation signaling, apoptosis, and chemotherapy response. It was broadly observed that MeCP2 functions as a proliferation and anti-apoptosis regulator while MBD1 is a negative regulator of invasion and migration. Moreover, gene chip microarray analysis confirmed unique transcriptional profiles, suggesting unique DNA methylation reader roles in prostate cancer (Yaqinuddin et al., 2008). MeCP2 supports androgen-independent proliferation in both normal epithelial and tumor-derived prostate cells by maintaining c-Myc levels. Although refractive to first-line bicalutamide antihormonal therapy, cells retain chemotherapy sensitivity regardless of *MECP2* status (Bernard et al., 2006). Building upon these results, *MECP2* loss-of-function studies resulted in increased senescence, apoptosis, and cells distributed in S phase. Altered nuclear lamina shape and nuclear rim along with depleted lamin proteins suggests MeCP2 supports cell division through nuclear envelope stability (Babbio et al., 2012). Regarding therapy response, MeCP2 may contribute to bicalutamide resistance through the miR-137- TRIM24 axis and docetaxel resistance through GADD45A transcriptional inactivation (Guan et al., 2019). DNMT inhibitor treatment reactivated *GADD45A* in the presence of MeCP2 and resulted in enhanced docetaxel sensitivity (Ramachandran et al., 2009). Additional publications confirmed MeCP2 occupancy at methylated *TIMP2*, *CCND2*, *NEUROG1*, *NRF2*, and *KAI1* promoters, however, the transcriptional and biological consequences remain unknown (Pulukuri et al., 2007; Müller et al., 2010; Shu et al., 2011; Khor et al., 2014; Lee et al., 2017). Lastly, LEDGF/p75, and its splice variant LEDGF/p52, are the only protein co-factors known to interact with and modulate MeCP2 in prostate cancer. This interaction results in cell context-specific activation of Hsp27 (Leoh et al., 2012). Future experiments could determine if this interaction broadly alters transcription, alternative splicing, and chromatin organization given prior LEDGF-MeCP2 publications (Li et al., 2016; Lesire et al., 2025). Looking ahead, investigating the clinical significance of MeCP2 in prostate biospecimens would garner evidence for future MeCP2 transcriptional and splicing regulation and co-factor functional validation.

#### 3.3.7 Glioma

Gliomas are the most common and malignant primary brain tumors with one of the worst survival rates (Weller et al., 2024). Limited clinical evidence has suggested a potential link for MeCP2 in glioma development. Patient-derived glioma tissues displayed elevated *MECP2* expression with low grade tumors having pronounced overexpression (Bian et al., 2019; Huang et al., 2023). Additional clinical studies could validate and strengthen these results using expanded public databases or by assessing clinicopathological features.

Limited cellular and mouse models link MeCP2 to glioma cancer hallmarks. Current publications present discordant results pertaining to MeCP2’s involvement in proliferation and epithelial-mesenchymal transition in gliomas. On one hand, the exogenous overexpression of MeCP2 reduces cell proliferation, migration, invasion, and adhesion (Sharma et al., 2018a). Conversely, other groups found that knockdown of MeCP2 also hinders *in vitro* and *in vivo* proliferation and mesenchymal markers while inducing apoptotic signaling and epithelial phenotypes (Bian et al., 2019; Huang et al., 2023). These studies provide additional mechanistic evidence for MeCP2 in mesenchymal induction. By interacting with SUV39H1, MeCP2 silences miR-200c expression and thus epithelial induction (Bian et al., 2019). Alternatively, downregulation of miR-138-5p releases MeCP2 from degradation and enhances mesenchymal-like behaviors and Wnt/β-catenin signaling (Huang et al., 2023). Although the functional implications remain to be determined, MeCP2 can differentially regulate myelin genes, suppress the TFPI-2 and CD133 methylated promoters, and interact with TET1 (Konduri et al., 2003; Gopisetty et al., 2013; Cartron et al., 2013; Sharma et al., 2018b). Future glioma studies can continue to decipher the clinical potential of MeCP2 by understanding its genomic distribution, co-factor identification and functionality, and regulation of transcription, splicing, and genomic stability.

#### 3.3.8 Lung cancer

Lung cancer is the leading cause of cancer-related deaths, accounting for 20% of patient deaths in 2024 (Siegel et al., 2024). Early studies using patient biopsies report *MECP2* upregulation in small-cell lung cancer and conflicting expression reports in non-small cell lung cancer (Müller-Tidow et al., 2001; Sato et al., 2002). Investigations using cell and mouse lung adenocarcinoma models are limited. MeCP2 may function during lung cancer proliferation as cell lines with high copy number were more susceptible to growth inhibition upon shRNA knockdown than cells with low copy number (Neupane et al., 2016). Reports also link MeCP2 occupancy at methylated promoters to transcriptional suppression of CEBPA, a tumor suppressor, and IGFBP-3, an apoptosis-inducer (Chang et al., 2004; Tada et al., 2006). In the case of IGFBP-3, promoter methylation also recruits SP-1 and HDAC and is rescued with DNMT inhibition (Chang et al., 2004). Additionally, Han and colleagues validated the LICN00518/miR-185-3p/MeCP2 axis in lung adenocarcinoma cell models. Elevated expression of LICN00518 was observed in tumor tissues, and knockdown abrogated *in vitro* and *in vivo* model proliferation. How MeCP2 mechanistically contributes to these phenotypes remains to be evaluated (Han et al., 2021). The present evidence suggests intriguing connections between MeCP2 and lung carcinomas; however, MeCP2 depletion studies would build upon this foundation and address the direct contribution of MeCP2 in this context.

#### 3.3.9 Renal cell carcinoma

Renal cell carcinoma is the most common form of kidney cancer (Rose and Kim, 2024). Clinical and preclinical studies indicate a potential function of MeCP2 in renal cell carcinoma. Two independent reports demonstrate low *MECP2* transcript levels correlate with poor overall survival and disease-free survival in patients (Liu H. et al., 2020; Gong et al., 2023). Furthermore, Gong and colleagues generated a clear cell renal cell carcinoma prognostic signature where *MECP2* served as a protective gene marker (Gong et al., 2023). Cell models confirmed that MeCP2 overexpression perturbed cell proliferation, migration, and invasion and is targeted by miR-454 (Liu H. et al., 2020). Additionally, MeCP2, along with MBD2, HDAC1, and mSin3a, associates with the methylated ABCG2 promoter resulting in gene inactivation which is restored through DNMT1 inhibition (To et al., 2006). The current literature suggests that MeCP2 plays a tumor suppressive role in renal cell carcinoma that would be bolstered with additional mechanistic validation.

#### 3.3.10 Cancers with understudied MeCP2 function

MeCP2 is implicated in additional cancer types beyond the examples described, but the current interpretation and understanding of these findings are limited. While investigating the mechanism of cisplatin resistance in melanoma, Chen and colleagues determined that tumors overexpress NEAT1 which releases MeCP2 inhibition through miR-519c-3p downregulation. Restoring miR-519c-3p sensitized a cisplatin-resistant NEAT1 overexpression cell model to chemotherapy (Chen et al., 2023). In esophageal squamous cell carcinoma models, the MAGE-A11 promoter undergoes methylation-dependent regulation. The hypermethylated promoter is bound by MeCP2 which recruits HDAC1 and HDAC2 for regional histone deacetylation. Additionally, transcriptional inactivation histone marks replace active marks to further enforce a suppressive state (Liu et al., 2018). In the case of osteosarcoma, the limited literature indicates that MeCP2 promotes *in vitro and in vivo* proliferation, invasion, and migration. These malignant properties may be due to its anti-apoptotic effect, localization to the TSSC3 gene, and/or loss of miR384 inhibition (Meng et al., 2014; Meng et al., 2015; Tan et al., 2020). Since MeCP2 binds to and regulates LANA-mediated suppression of Kaposi’s sarcoma-associated herpesvirus (KSHV), how MeCP2 contributes to the KSHV lifecycle may be of interest (Matsumura et al., 2010). Studies investigating myeloid and lymphoid leukemia lineages observe MeCP2 transcript and protein overexpression in patient samples, along with MeCP2-linked gene regulation (Kn et al., 2004; Zhang X. et al., 2017; Li Z. et al., 2018). For example, in acute myelogenous leukemia, the AML1-ETO fusion transcription factor epigenetically silences Bcl-2, CEBPA and p14(ARF) genes by recruiting MeCP2 and enforcing a repressive histone PTM state (Zhuang et al., 2013). Whereas in advanced chronic myeloid leukemia, the overexpression of lncRNA MEG3 and miR-147 reduces the expression of MeCP2 and other DNA methylation modulators suggesting a mode of non-coding RNA regulation (Li Z. et al., 2018). Although MeCP2 binds the *MDR1* or *CEBPD* methylated promoters in acute lymphoblastic or myeloid leukemia, respectively, the functional consequences of this localization remain unknown (El-Osta et al., 2002; Agrawal et al., 2007). For uterine, cervical, and head and neck cancers, *MECP2* amplification and *RAS* family oncogene alterations are mutually exclusive, suggesting MeCP2 and RAS mutations are functionally redundant (Neupane et al., 2016). Cervical cancer tissues and cell models expressed reduced *MECP2* and *MBD2* which could be restored miR-221/222 depletion (Pan et al., 2019). On the other hand, in oral cavity squamous cell carcinoma, the most common type of head and neck cancer, *MECP2* was upregulated in carcinoma tissues and enhanced markers of cell proliferation and Wnt/β-Catenin signaling while dampening pro-apoptotic markers. These phenotypes were reversed when miR-106a exogenous expression inhibited MeCP2 (Zhang et al., 2018). While these reports expand our understanding of MeCP2 in various cancer types, much more work is needed to evaluate the role of MeCP2 across diverse tumor types and address knowledge gaps in translational models.

## 4 Discussion

The evidence presented in this review highlights the latest findings in MeCP2 regulation, function, and potential mechanisms of action in the settings of cancer and immune homeostasis. Proper function and dosage of MeCP2 are critical for postnatal brain and peripheral organ health with implications extending beyond MeCP2-pathologies historically defined by neurological symptoms. Continuing to understand MeCP2 regulation at the translational and alternative splicing levels may continue to reveal actionable therapeutic avenues for MeCP2-pathologies. MeCP2 function is multilayered with co-factor partnering and underlying DNA methylation modification, pattern, and location within genomic elements informing contextual consequences targeting multiple epigenic levels. As MeCP2 is an integral hub at the junction of DNA methylation, protein, and RNA signaling interactions, it will be informative for research groups to take as many layers as possible into account when studying their gene or biological process of interest to understand MeCP2 function in its holistic context. Along these lines, MeCP2 PTMs are crucial for mediating protein co-factor interactions and downstream function, primarily exemplified through phosphorylation studies. PTMs may play a key role as a molecular switch dictating how MeCP2 executes its myriad epigenetic functions. Extending this knowledge to additional PTMs, including methylation, acetylation, SUMOlyation, lactylation, and glycosylation, and investigating the dynamics between these PTMs and RNA co-factors would fill current knowledge gaps in the field.

Beyond MeCP2-driven neurological disorders, MeCP2 is also a key epigenetic regulator of cell maintenance and function in the immune system and cancer settings. MeCP2 is involved in the regulation of T cells, natural killer, macrophages, and non-immune cells where it appears to function as a transcriptional regulator of cell surface and secreted immunomodulatory proteins. This contributes to immune imbalance through altered immune populations, elevated inflammatory conditions, and increased risk of autoimmune diseases. Whether the dynamic and complex nature of MeCP2 directly contributes to the development and function of other immune cell types within the tumor microenvironment remains a major knowledge gap. Within cancer models, the contextual nature of MeCP2 can support or inhibit malignancy through direct or indirect transcriptional control. This variation may arise from cancer type specific oncogenic drivers, mutational landscapes, and molecular pathways which influence MeCP2 expression, PTM regulation, and available co-factors, for example. Looking ahead, expanding the use of MeCP2 loss-of-function studies and unbiased profiling using next-generation sequencing (i.e., RNA-Seq, ChIP-Seq, ATAC-Seq) technologies will assist to comprehensively map and assess the functional significance of MeCP2 beyond select gene panels and standard cancer hallmark phenotypes. Research groups may consider looking beyond transcriptional regulation to explore the role of MeCP2 PTMs and alternative splicing, proteome, and chromatin organization regulation. Finally, given the link between MeCP2 and patient immune imbalance, it would be prudent to characterize how altered MeCP2 tumor expression, function, or co-factors influence the immune microenvironment.
